# Risk for Cardiovascular Adverse Events Associated With Sphingosine-1-Phosphate Receptor Modulators in Patients With Multiple Sclerosis: Insights From a Pooled Analysis of 15 Randomised Controlled Trials

**DOI:** 10.3389/fimmu.2021.795574

**Published:** 2021-12-07

**Authors:** Zhao Zhao, Yang Lv, Zhi-Chun Gu, Chun-Lai Ma, Ming-Kang Zhong

**Affiliations:** ^1^ Department of Pharmacy, Huashan Hospital, Fudan University, Shanghai, China; ^2^ The First Affiliated Hospital, College of Clinical Medicine of Henan University of Science and Technology, Luoyang, China; ^3^ Department of Pharmacy, Ren Ji Hospital, Shanghai Jiao Tong University School of Medicine, Shanghai, China

**Keywords:** multiple sclerosis, Sphingosine 1-phosphate receptor modulators, cardiovascular adverse events, meta-analysis, bradyarrhythmia, hypertension

## Abstract

**Background:**

All agents engaging sphongosine-1-phospate receptors (S1PRs) will have some cardiovascular effect. This study aimed to elucidate the risk of cardiovascular adverse events (AEs) in patients with multiple sclerosis (MS) treated with S1PR modulators (S1PRMs).

**Methods:**

We systematically searched the PubMed, EMBASE, and Cochrane Library databases for randomised controlled trials (RCTs) published through January 5, 2021. Relative risks (RRs) and 95% confidence intervals (CIs) were calculated using the random-effects model. Sensitivity analyses and meta-regression were performed.

**Results:**

Seventeen RCTs (12 for fingolimod; 3 for ozanimod; 2 for siponimod) involving 13,295 patients were included. Compared with the control treatment, S1PRMs significantly increased the risk of cardiovascular AEs (RR, 2.21; 95% CI, 1.58–3.10; I^2^, 75.6%). Notably, the high-risk cardiovascular AEs associated with S1PRMs were primarily bradyarrhythmia (RR, 2.92; 95% CI, 1.91–4.46; I^2^, 30.8%) and hypertension (RR, 2.00; 95% CI, 1.49–2.67; I^2^, 56.5%). Subgroup analysis results were consistent with the primary outcomes except that ozanimod was associated with a higher risk of hypertension only (RR, 1.76; 95% CI, 1.10–2.82; I^2^, 0.0%), while siponimod was associated with a higher risk of bradyarrhythmia only (RR, 2.75; 95% CI, 1.75–4.31; I^2^, 0.0%). No significant inter-subgroup differences were observed (P_interaction_ > 0.05).

**Conclusions:**

S1PRM use increased the risk of cardiovascular AEs by 1.21 times in patients with MS, and increased risks for bradyarrhythmia and hypertension were at 2.92- and 2.00-fold, respectively. These findings can help clinicians assess the risk of cardiovascular AEs in patients treated with S1PRMs.

**Systematic Review Registration:**

The PROSPERO ID is CRD42020183215.

## Introduction

Multiple sclerosis (MS) is a chronic autoimmune disease of the central nervous system (CNS) that is mainly characterised by inflammatory demyelination, leading to severe neurological disability (1). MS typically affects young adults and is commonly diagnosed in the early years by acute relapses, followed by a partial or complete remission period (1). The clinical course of MS is characterised by 3 major classifications: relapsing-remitting, primary progressive, and secondary progressive (2). There is currently no definite cure for MS, but a number of disease-modifying treatments (DMTs) that mainly target inflammatory settings have been designed to reduce the recurrence rate and the accumulation of disability in MS patients (3). Sphingosine-1-phosphate receptor modulators (S1PRMs), a class of DMTs, have a structure similar to sphingosine or sphingosine-1-phosphate (S1P), a bioactive lysophospholipid abundant in erythrocytes, the brain, the spleen, and the eyes that regulates diverse cellular responses involved in the immune system, the CNS, the blood–brain barrier, and the cardiovascular system through 5 distinct G protein–coupled receptor subtypes (S1PR_1_ to S1PR_5_) (3–5). Previous and continuing investigations have revealed the positive effects of the S1P pathway and S1PRMs on immune cell trafficking and neuroprotection, leading the approval of 3 S1PRMs to treat MS, including fingolimod (FTY720, Gilenya^®^), siponimod (BAF312, Mayzent^®^), and ozanimod (RPC1063, Zeposia^®^) (5). Several other agents are involved in the clinical development of MS, including amiselimod, ceralifimod, etrasimod, and GSK2018682 (5–7).

Due to extensive S1PR expression on cardiomyocytes and vascular endothelial cells, all agents that engage them will have some cardiovascular effect (4, 8–10). As the first approved S1PRM, fingolimod non-selectively targets S1PR_1_, S1PR_3_, S1PR_4_, and S1PR_5_ (6). Several cardiovascular adverse events (AEs) of fingolimod, such as atrioventricular block, sinus bradycardia, orthostatic hypotension, and hypertension, have been reported (8). In three phase 3 clinical trials of fingolimod, a transient decrease in mean measured heart rate occurred 4–5 h after the first dose, with a maximum reduction of 8–11 beats per minute below baseline (11). Small mean increases in systolic and diastolic blood pressure were observed with long-term treatment in fingolimod clinical trials, and increases in blood pressure were apparent at 2 months after starting therapy and increased over the following 4 months before stabilising (8, 11). Since studies have shown that such cardiovascular effects might be mediated by transient agonism of S1PR_1_ and/or S1PR_3,_ second-generation S1PRMs with low affinity for S1PR_3_ have been extensively developed (5). However, similar cardiovascular AEs were observed in MS patients receiving siponimod, ozanimod, and other second-generation agents that minimise S1PR_3_ binding (7, 9, 12–14). At present, for the approved S1PRMs, 6-h continuous cardiac monitoring after the first dose and dose titration are recommended to reduce these side effects to a certain extent (14–16). Nevertheless, no study to date has elucidated the risk of cardiovascular AEs in patients with MS treated with S1PRMs. Therefore, this study summarised all available evidence from randomised controlled trials (RCTs) to provide a comprehensive and rigorous systematic review that explores: 1) the relative risks (RRs) of cardiovascular AEs in MS patients treated with approved S1PRMs; 2) the types of cardiovascular AEs that carry a relatively high risk; and 3) whether the risk varies by S1PRM used and dosage versus control treatments.

## Method

### Literature Search and Study Selection

We followed the standards of the Cochrane Collaboration and the Preferred Reporting Items for Systematic Reviews and Meta-Analyses statement for reporting systematic reviews (17). We searched the PubMed, EMBASE, and Cochrane Library databases for RCTs published in English through January 5, 2021 of S1PRM treatment in patients with MS. Full details of the search items are presented in **Supplementary Table 1**. We also identified potential studies from the ClinicalTrials.gov platform (www.clinicaltrials.gov). Studies that compared S1PRMs with a placebo or other disease-modifying treatments (DMTs; interferon-β, glatiramer acetate, teriflunomide, dimethyl fumarate, natalizumab, alemtuzumab, ocrelizumab, daclizumab, mitoxantrone, etc.) in patients with MS and reported data on cardiovascular AEs were eligible for inclusion. Two reviewers (ZZ and YL) independently screened all citations from the initial search. Any discrepancies were resolved *via* consultation with a third reviewer (ZG).

### Study Outcomes and Data Extraction

The study outcomes were the overall cardiovascular AEs (including general and serious cardiovascular AEs) and specified cardiovascular AEs (including any arrhythmia, bradyarrhythmia, tachyarrhythmia, hypertension, hypotension, heart failure, coronary artery disease, acute coronary syndrome, and chronic coronary syndrome). Cardiovascular AEs were determined as being possibly or quite likely related to the study drug as assessed by the investigators. According to the ClinicalTrials.gov platform (www.clinicaltrials.gov), serious cardiovascular AEs were defined as those with the following results: (1) life-threatening or fatal; or (2) patient hospitalisation or extension of a current hospital stay resulting in an ongoing or significant incapacity or substantial interference with normal life function. A cardiovascular AE that did not meet this definition above was considered a general cardiovascular AE. Arrythmia encompasses bradyarrhythmia and tachyarrhythmia. Bradyarrhythmia included bradycardia, sinus bradycardia, and first- through third-degree atrioventricular blocks. Tachyarrhythmia included tachycardia, paroxysmal tachycardia, ventricular tachycardia, sinus tachycardia, supraventricular tachycardia, ventricular extrasystoles, supraventricular extrasystoles, palpitations, cardiac flutter, atrial fibrillation, and ventricular fibrillation. Hypertension also included secondary hypertension and hypertensive crisis, while hypotension also included orthostatic hypotension. Heart failure included stress cardiomyopathy, left ventricular dysfunction, hypertensive heart disease, mitral valve incompetence, and tricuspid valve incompetence. Coronary artery disease encompassed acute coronary syndrome and chronic coronary syndrome. Acute coronary syndrome also included coronary artery thrombosis, coronary artery occlusion, unstable angina, and myocardial infarction. Chronic coronary syndrome included coronary artery stenosis, angina pectoris, and myocardial ischaemia. Data were extracted independently by 2 authors (ZZ and YL) using an a priori–designed form that included the study characteristics, patient demographics, and clinical characteristics. Data on cardiovascular AEs were collected for each of the studies, including the occurrence number and total number of published RCTs and ClinicalTrials.gov platform.

### Quality Assessment

The methodological quality of each RCT was evaluated using the Cochrane Collaboration Risk of Bias Tool as low, unclear, or high in terms of the risk of bias based on the following domains: random sequence generation (selection bias), allocation concealment (selection bias), blinding (performance bias and detection bias), incomplete outcome data (attrition bias), and selective reporting (reporting bias) (18).

### Data Analysis

Forest plots were used to measure the outcomes, and RRs and their 95% confidence intervals (CIs) were used to calculate the comparative effect sizes with random-effects models. Interstudy heterogeneity was assessed using the I^2^ statistic (values of I^2^ > 50% considered significant) (19). Prespecified subgroup analyses were performed according to cardiovascular AE severity (general or serious), S1PRM(s) used (fingolimod, ozanimod, and siponimod), and S1PRM dosage (1.25 vs. 0.5 mg/d for fingolimod, 1.0 vs. 0.5 mg/d for ozanimod) versus controls (placebo and other DMTs). Interaction analyses (P_interaction_) were conducted to evaluate the comparability of the outcomes within each subgroup. A leave-one-out sensitivity analysis was applied to each meta-analysis to detect the robustness of the results. Because potential effect modifiers (patient demographics and clinical characteristics) may lead to outcome bias, a meta-regression analysis was performed to address the influence of these factors on the risk of cardiovascular AEs. As a rule, at least 25% of data points should be available for each variable in a meta-regression (20). Potential publication bias was evaluated by visual inspection of the funnel plots and quantitative analysis using the Begg’s and Egger’s tests (21). The trim and fill method was used to manage publication bias (22). This method requires no assumptions about the mechanism that leads to publication bias and provides an estimate of the number of missing studies as well as an estimated intervention effect to adjust for it. Statistical analyses were performed using STATA software (version 12.0; STATA Corporation), with values of P < 0.05 considered statistically significant.

## Results

### Search Results and Study Evaluation

**Figure 1:**
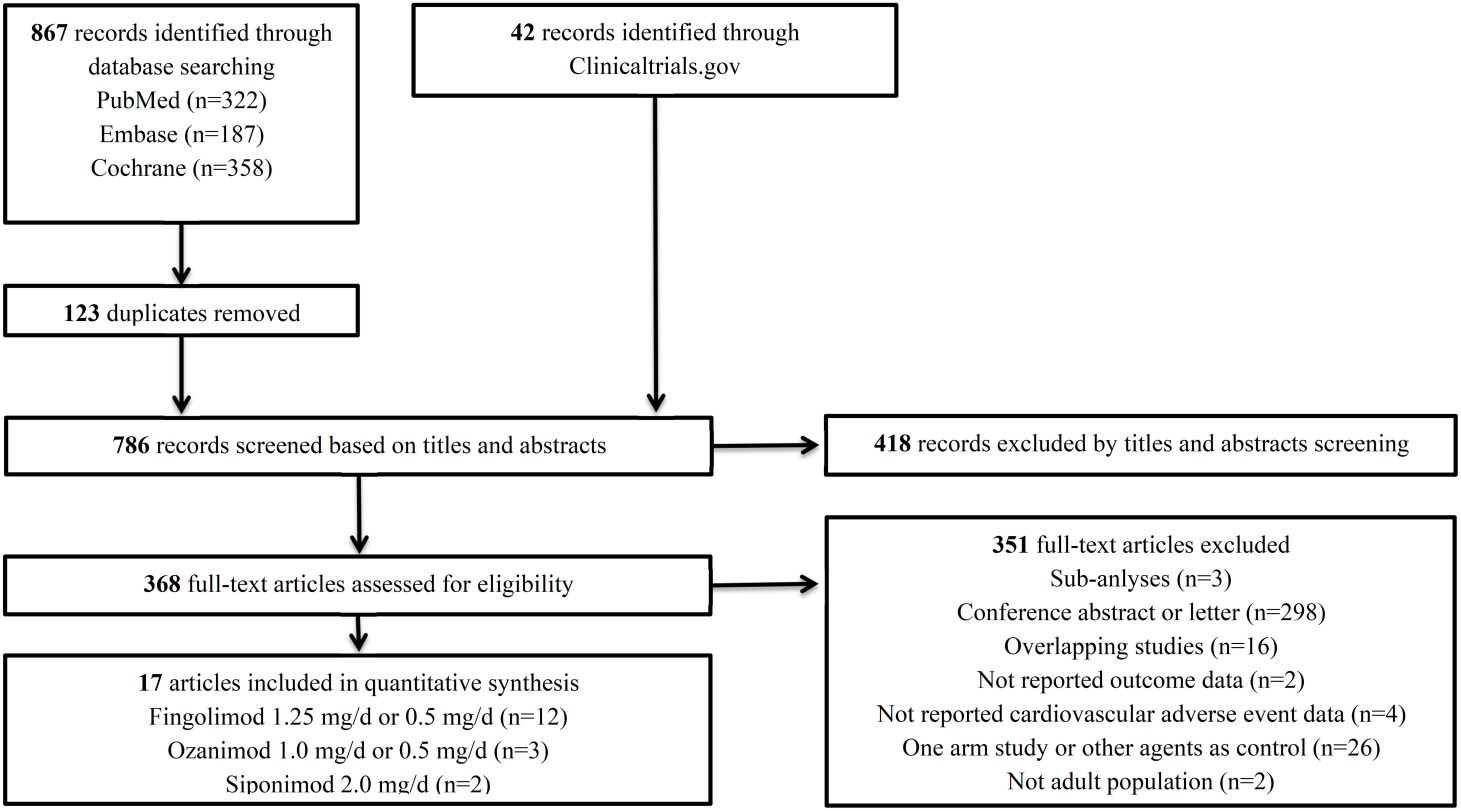
Flow diagram for the selection of eligible studies.

Our initial search identified 867 articles from the databases and 42 records from the ClinicalTrials.gov platform; 123 duplicates were removed, while another 786 articles were excluded by the title and abstract screening. We reviewed the full text of the remaining 368 articles and ultimately included 17 RCTs of 3 S1PRMs (12 RCTs for fingolimod 1.25 mg/d or 0.5 mg/d; 3 RCTs for ozanimod 1.0 mg/d or 0.5 mg/d; 2 RCTs for siponimod 2 mg/d) (**Figure 1**). The detailed characteristics of the included trials are presented in **Table 1** and **Supplementary Table 2**. The included studies were published between 2006 and 2020 with trial durations of 6–36 months. A total of 13,295 patients were enrolled, among which 8,157 were treated with S1PRMs and 5,138 were treated with a placebo or other active DMT (interferon beta, glatiramer acetate, or natalizumab). The details of the quality evaluation are summarised in **Supplementary Table 2**. Of the 17 trials, 12 (12/17, 70.6%) had a low risk of bias, among which 7 fully met all items of the quality assessment, 2 had an unclear risk in terms of a random sequence generation and incomplete outcome data, and 1 had a high risk in terms of allocation concealment and blinding of participants and personnel.

**Table 1:** Characteristics of included studies and baseline characteristics of the population included in the RCTs.

Source/Study	NCT	Disease type	Interventions/Arms	Duration, month	Patients, No.
Kappos et al., 2006 (23) (FTY720 D2201)	NCT00333138	RRMS/SPMS	fingolimod 1.25 mg/d	6	94
			placebo		93
Cohen et al., 2010 (24) (TRANSFORMS)	NCT00340834	RRMS	fingolimod 1.25 mg/d	12	420
			fingolimod 0.5 mg/d		429
			interferon beta-1a 30 μg/w		431
Kappos et al., 2010 (25) (FREEDOMS)	NCT00289978	RRMS	fingolimod 1.25 mg/d	24	429
			fingolimod 0.5 mg/d		425
			placebo		418
Saida et al., 2012 (26)	NCT00537082	RRMS/SPMS	fingolimod 1.25 mg/d	6	54
			fingolimod 0.5 mg/d		57
			placebo		57
Selmaj et al., 2013 (12) (BOLD)	NCT00879658	RRMS	siponimod 2 mg/d	6	49
			placebo	6	61
Calabresi et al., 2014 (15) (FREEDOMS II)	NCT00355134	RRMS	fingolimod 1.25 mg/d	24	370
			fingolimod 0.5 mg/d		358
			placebo		355
Fox et al., 2014 (27) (EPOC)	NCT01216072	RRMS	fingolimod 0.5 mg/d	6	783
			iDMT (interferon beta or glatirameracetate)		245
Cohen et al., 2016 (28) (RADIANCE)	NCT01628390	RRMS	ozanimod 0.5 mg/d	6	87
			ozanimod 1.0 mg/d		83
			placebo		88
Lublin et al., 2016 (29) (INFORMS)	NCT00731692	PPMS	fingolimod 1.25 mg/d	36	147
			fingolimod 0.5 mg/d		336
			placebo		487
Popova et al., 2017 (30) (GIMN)	NR	RRMS	fingolimod 0.5 mg/d	6	230
			interferon beta-1a 44 μg or glatirameracetate		68
Comi et al., 2017 (31) (GOLDEN)	NCT01333501	RRMS	fingolimod 0.5 mg/d	18	104
			interferon beta-1b 250 µg every other day		47
Kappos et al., 2018 (14) (EXPAND)	NCT01665144	SPMS	siponimod 2 mg/d	36	1099
			placebo		546
Cree et al., 2018 (32) (PREFERMS)	NCT01623596	RRMS	fingolimod 0.5 mg/d	12	433
			iDMT (interferon beta or glatirameracetate)		428
Cohen et al., 2019 (33) (RADIANCE)	NCT02047734	RRMS/SPMS/PPMS	ozanimod 0.5 mg/d	24	439
			ozanimod 1.0 mg/d		434
			interferon beta-1a 30µg/w		440
Comi et al., 2019 (16) (SUNBEAM)	NCT02294058	RRMS/SPMS/PPMS	ozanimod 0.5 mg/d	12	453
			ozanimod 1.0 mg/d		448
			interferon beta-1a 30µg/w		445
Butzkueven et al., 2020 (34) (REVEAL)	NCT02342704	RRMS	fingolimod 0.5 mg/d	13	54
			Natalizumab 300 mg/4w		54
Cree et al., 2020 (35) (ASSESS)	NCT01633112	RRMS	fingolimod 0.5 mg/d	12	345
			glatirameracetate 20 mg/d s.c.		324

iDMT, injected disease-modifying therapy; RRMS, relapsing-remitting multiple sclerosis; PPMS, primary progressive multiple sclerosis; SPMS, secondary progressive multiple sclerosis; sc, subcutaneous; NR, not reported; NCT, National Clinical Trial.

### Risk of Cardiovascular AEs Reported for S1PRMs

#### Overall Cardiovascular AEs

**Figure 2:**
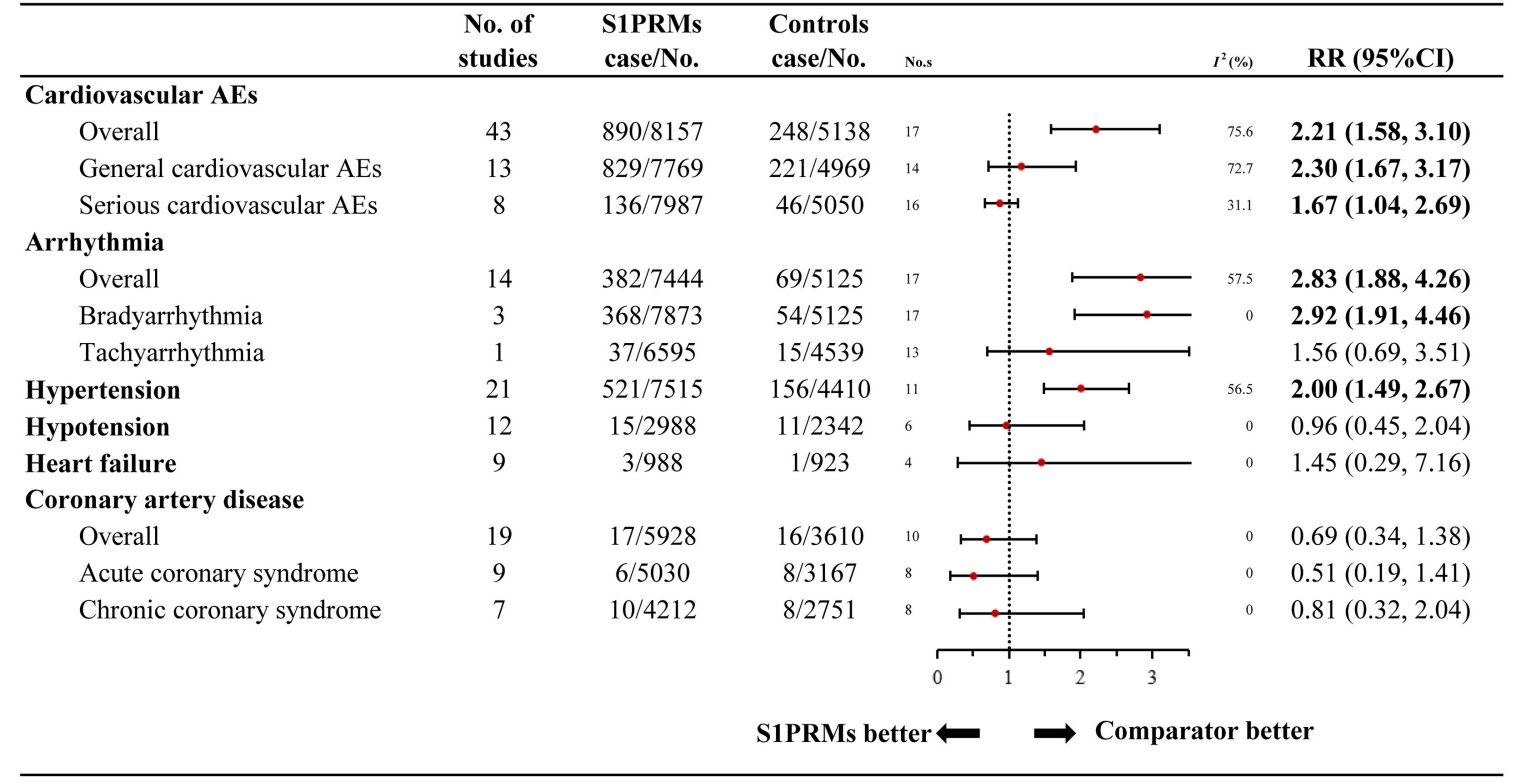
Relative risk of cardiovascular adverse events reported for S1PRMs in comparison to the control treatment. AEs, adverse events; I^2^, heterogeneity; No. s, numbers of studies; RR, relative risk; 95% CI, 95% confidence interval; S1PRMs, sphingosine-1-phosphate receptor modulators.

The results of the overall cardiovascular AEs are summarised in **Figure 2**. The overall rate of cardiovascular AEs was 10.9% (890/8,157) in the S1PRM-treated group versus 4.8% (248/5,138) in the control group, indicating a significantly higher risk of cardiovascular AEs in patients treated with S1PRMs than in those receiving the control treatment (RR, 2.21; 95% CI, 1.58–3.10; I^2^, 75.6%). The subgroup analysis indicated that general (RR, 2.30; 95% CI, 1.67–3.17; I^2^, 72.7%) and serious (RR, 1.67; 95% CI, 1.04–2.69; I^2^, 31.1%) cardiovascular AEs were significantly more prevalent in the S1PRM treatment group than in the control group (P_interaction_ > 0.05).

#### Specified Cardiovascular AEs

The 9 categories of specified cardiovascular AEs are outlined in **Figure 2**. All outcomes presented low heterogeneity except for hypertension (I^2^ = 56.5%). No significant differences were found between the S1PRM-treated group and the control group in terms of hypotension (RR, 0.96; 95% CI, 0.45–2.04; I^2^, 0.0%), heart failure (RR, 1.45; 95% CI, 0.29–7.16; I^2^, 0.0%), coronary artery disease (RR, 0.69; 95% CI, 0.34–1.38; I^2^, 0.0%), acute coronary syndrome (RR, 0.51; 95% CI, 0.19–1.41; I^2^, 0.0%), and chronic coronary syndrome (RR, 0.81; 95% CI, 0.32–2.04; I^2^, 0.0%). Compared with the control treatment, S1PRM use significantly increased the risk of arrhythmia (RR, 2.83; 95% CI, 1.88–4.26; I^2^, 42.4%) and hypertension (RR, 2.00; 95% CI, 1.49–2.67; I^2^, 56.5%). Notably, the increased risk for arrhythmia associated with S1PRM use was primarily bradyarrhythmia (RR, 2.92; 95% CI, 1.91–4.46; I^2^, 30.8%) rather than tachyarrhythmia (RR, 1.56; 95% CI, 0.69–3.51; I^2^, 25.7%).

**Figure 3:**
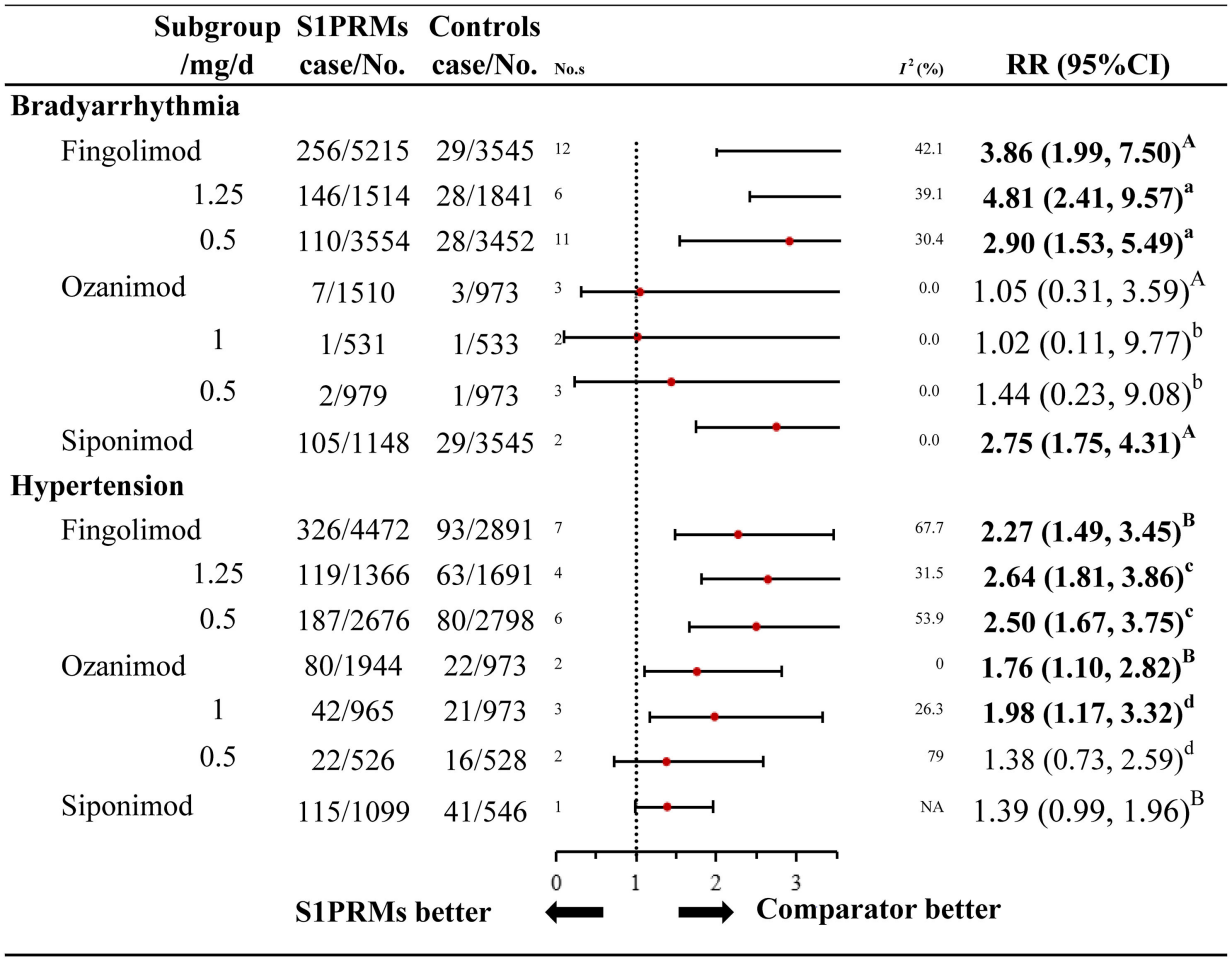
Subgroup analysis of high-risk cardiovascular adverse events of different S1PRMs. ^A^, for individual S1PRMs in bradyarrhythmia, P_interaction_ = 0.140; ^B^, for individual S1PRMs in hypertension, for P_interaction_ = 0.266; ^a^, for different doses of fingolimod in bradyarrhythmia, P_interaction_ = 0.360; ^b^, for different doses of ozanimod in bradyarrhythmia, P_interaction_ = 0.851; ^c^, for different doses of fingolimod in hypertension, P_interaction_ = 0.900; ^d^, for different doses of ozanimod in hypertension, P_interaction_ = 0.408; NA, not applicable; I^2^, heterogeneity; No. s, numbers of studies; RR, relative risk; 95% CI, 95% confidence interval; S1PRMs, sphingosine-1-phosphate receptor modulators; DMTs, disease modulating therapies.

For high-risk cardiovascular AEs (bradyarrhythmia and hypertension), we further performed subgroup analyses to explore whether these risks varied among the different individual S1PRMs, and doses versus the control treatments (**Figure 3** and **Supplementary Table 6**). As for the different individual S1PRMs, fingolimod was associated with an increased risk of both bradyarrhythmia (RR, 3.86; 95% CI, 1.99–7.50; I^2^, 42.1%) and hypertension (RR, 2.27; 95% CI, 1.49–3.45; I^2^, 67.7%). Ozanimod was associated with a higher risk of hypertension (RR, 1.76; 95% CI, 1.10–2.82; I^2^, 0.0%), while siponimod was associated with a higher risk of bradyarrhythmia (RR, 2.75; 95% CI, 1.75–4.31; I^2^, 0.0%). However, we failed to find an estimated difference among the different individual S1PRMs (P_interaction_ = 0.140). Fingolimod and ozanimod were available in 2 doses each (0.5 mg/d and 1.25 mg/d versus 0.5 mg/d and 1.0 mg/d, respectively), while siponimod was available in one dose (2.0 mg/d). The results of fingolimod were also in accordance with the primary analyses regarding both categories of high-risk cardiovascular AEs with no significant difference between the 0.5 mg/d and 1.25 mg/d groups (P_interaction_ = 0.360 for bradyarrhythmia; P_interaction_ = 0.851 for hypertension). Compared with the control treatments, ozanimod 1.0 mg/d carried a significantly higher risk of hypertension (RR, 1.98; 95% CI, 1.17–3.32; I^2^, 0.0%), a finding that was consistent with the primary analyses. No significant difference was found in the risk of hypertension between the ozanimod 0.5 mg/d and control treatments (RR, 1.38; 95% CI, 0.73–2.59; I^2^, 0.0%). However, we found no estimated difference between the different doses of ozanimod (P_interaction_ = 0.900). As for the different control treatments, the overall results for the high-risk cardiovascular AEs were also consistent with the primary analyses, and no significant differences were found between the group with placebo as the control and that with other DMTs as the control (P_interaction_ = 0.445 for bradyarrhythmia; P_interaction_ = 0.225 for hypertension).

### Sensitivity Analyses and Meta-Regressions

Sensitivity analyses failed to identify any individual trial as having influenced the outcomes, thus confirming the robustness of these results (**Supplementary Table 4**). Twelve potential effect modifiers, including patient demographics (age and sex) and clinical characteristics (Expanded Disability Status Scale scores, disease duration, relapse in the previous 1 year, relapse in the previous 2 years, number of patients without G+ lesions on T1-weighted images, number of G+ lesions on T1-weighted images, volume of lesions on T2-weighted images, normalised brain volume, and number of patients given previous treatment), were assessed in the meta-regression, and the results suggested that the incidence of cardiovascular AEs was not associated with any of the aforementioned factors (**Supplementary Table 5**).

### Publication Bias

The funnel plots for trials reporting general cardiovascular AEs, arrhythmia, bradyarrhythmia, and hypertension were all asymmetrical on visual inspection, and the corresponding P values for Egger’s test were 0.011, 0.010, 0.061, and 0.014, respectively (**Supplementary Figures 2, 4, 5, 8**), and the trim and fill method was applied to manage publication bias (**Supplementary Table 7**). The funnel plots for trials reporting cardiovascular AEs, serious cardiovascular AEs, tachyarrhythmia, and coronary artery disease were fairly symmetrical on visual inspection, suggesting that publication bias was not a concern (**Supplementary Figures 1, 3, 6, 7**). Because of the limited number of studies with hypotension (n = 6), heart failure (n = 4), acute coronary syndrome (n = 8), and chronic coronary syndrome (n = 8), funnel plots were not created for this purpose.

## Discussion

### Major Findings

This systematic review and meta-analysis is the first to provide a comprehensive overview of the risk of cardiovascular AEs associated with S1PRM use based on 17 RCTs of 13,295 MS patients. The major findings were as follows: 1) S1PRM use increased the risk of cardiovascular AEs by 1.21 times in MS patients, and the incidence for both general and serious cardiovascular AEs increased significantly; 2) patients treated with S1PRMs were at 2.92- and 2.00-fold increased risk for bradyarrhythmia and hypertension, respectively; 3) the risk for bradyarrhythmia and hypertension associated with S1PRMs might not change with S1PRM type or dose versus control treatment in patients with MS.

### Comparison With Previous Studies

No meta-analysis to date has specifically addressed the clinical cardiovascular AEs associated with S1PRM use. Only one meta-analysis (36) of 13 RCTs and 10,554 patients compared the overall side effects of S1PRMs and found that ozanimod was the best intervention with the highest acceptability (ozanimod 1 mg/d vs placebo: RR, 0.81; 95% CI, 0.28–2.33). With regard to the risk of arrhythmia induced by S1PRM use, our meta-analysis indicated that S1PRMs mainly increased the risk for bradyarrhythmia, a finding that is consistent with the results of several previous studies. One meta-analysis (37) published in 2020 evaluated the efficacy and safety of fingolimod in 10 RCTs and indicated that fingolimod use was associated with a significantly increased risk of bradycardia (RR, 3.92; 95% CI, 1.30–11.84), a finding that is consistent with our study. However, only bradycardia data were retrieved from that study, as its assessment of the risk of cardiovascular AEs was not comprehensive. Previous observational studies evaluated the cardiac effects of S1PRMs in clinical practice, primarily fingolimod, the first approved DMT. In 3 real-world open-label single-arm studies (38–41) focusing on cardiac AEs in patients treated with fingolimod, most cardiac AEs were bradyarrhythmia, including bradycardia, second-degree atrioventricular block, and 2:1 atrioventricular block; most AEs were asymptomatic, occurred within 6 h after the first dose, and were self-limiting. Consistent with the results of the aforementioned studies, bradycardia and atrioventricular block were observed in 2 retrospective database studies (42, 43) that examined the first-dose safety of fingolimod in clinical practice and 1 retrospective study (44) that considered its long-term cardiac safety. Approximately one-third of patients in these studies developed bradyarrhythmia; 3 needed intensive care unit treatment for a right bundle branch block or second-degree atrioventricular block, and only 1 had atrial fibrillation that required treatment after an average of 1.5 year of follow-up.

For the changes in blood pressure caused by S1PRMs, the current analysis of the AEs related to blood pressure in the included RCTs indicated that S1PRM use increased the risk of hypertension in MS patients. However, few studies focused on the effects of S1PRMs on blood pressure in clinical practice. One analysis specifically examined first-dose effects in the phase 3 studies and reported a small decrease in blood pressure in fingolimod-treated patients on day 1 that peaked at 4–5 h after the first dose (8). In contrast, small mean increases in systolic and diastolic blood pressures were observed with long-term fingolimod treatment, and increases in blood pressure were apparent at 2 months after starting therapy and then increased over the follow-up of 4 months to 1.5 years (8, 44). Similarly, the current meta-analysis also failed to find a relatively higher risk of hypotension for any S1PRM and suggested a significantly increased risk of hypertension. These findings indicated that a transient decrease in blood pressure might occur after the first dose, followed by a persistent increase in blood pressure with prolonged treatment. Moreover, in the long run, the incidence of hypertension might be higher than that of hypotension.

In terms of cardiovascular risk among the individual S1PRMs, the results of this analysis indicate that fingolimod use was associated with a higher risk of both bradyarrhythmia and hypertension than ozanimod or siponimod. Moreover, no significantly increased risks for bradyarrhythmia or hypertension were associated with ozanimod and siponimod use, respectively, compared to control, indicating that ozanimod might have a better safety profile for bradyarrhythmia, while siponimod might have a better safety profile for hypertension. Nevertheless, our analysis failed to find a significant difference among individual S1PRMs (P_interaction_ = 0.360 for bradyarrhythmia; P_interaction_ = 0.851 for hypertension). Only one study (45) compared the safety of fingolimod and ozanimod by matching the adjusted indirect data using the data from 5 large-scale phase 3 RCTs (TRANSFORMS, FREEDOMS, and FREEDOMS II trials for fingolimod, RADIANCE-B and SUNBEAM trials for ozanimod) and demonstrated that ozanimod was associated with significantly lower rates of conduction abnormalities and a significantly lower reduction in blood pressure versus fingolimod at the first dose as well as a significantly lower risk of bradycardia compared with fingolimod in the 2-year safety outcomes, which support the findings of this analysis. However, considering that all of these results were from RCTs, more real-world studies are needed to reinforce them. Our stratified analyses demonstrated that S1PRMs did not have dose-dependent effects. One meta-analysis (46) published in 2021 that evaluated the efficacy and safety of different doses of fingolimod by examining 11 RCTs found that fingolimod 0.5 mg/d showed a significantly higher risk for overall AEs (RR, 1.25; 95% CI, 1.01–1.54), but no significant difference was found between the 0.5 mg/d fingolimod group and the control treatment group in serious AEs including bradycardia (RR, 2.97; 95% CI, 0.75–11.72) and atrioventricular block (RR, 2.03; 95% CI, 0.45–9.25). In our study, regardless of AE severity, fingolimod 0.5 mg/d and 1.25 mg/d were associated with a significant high risk for bradyarrhythmia consisting of bradycardia and atrioventricular block without a statistically significant difference (P_interaction_ = 0.360). Although the results of bradycardia and atrioventricular block in that study did not reach statistical significance compared to this study, mainly due to the relatively small number of cases, the RRs that study tended to differ between the fingolimod and control treatment groups, which is broadly consistent with our findings.

### Potential Mechanism

Most S1PRMs in clinical development or approved for MS treatment display high affinity for S1PR1 and typically one or more of the other S1PR subtypes (5). Specifically, some S1PRMs, including fingolimod, are pro-drugs that must be phosphorylated by sphingosine kinases before they display high binding affinity for S1PRs (5). Several second-generation S1PRMs, such as siponimod and ozanimod, have been designed to not require phosphorylation for activity and increase their selectivity for S1PR1 (4, 5). The cardiovascular side effects of S1RPMs and the cardiovascular risk differences among individual S1PRMs can be explained by the wide distribution of S1PR1-5 (5, 47). The S1PR1 subtype is found in lymphocytes, cells of the CNS, atrial myocytes, and endothelial cells; S1PR2 and S1PR3 have similar expressions on CNS, endothelial, and smooth muscle cells; S1PR4 is mainly expressed on lymphocytic and haematopoietic cells but has also been detected on human airway smooth muscle cells; and S1PR5 is mainly expressed on oligodendrocytes in the CNS as well as on natural killer cells and other lymphocytes in the spleen (4).

Pre-clinical studies suggested that S1PR1 and S1PR3 could be the main candidates for S1PRM–induced cardiovascular AEs (5). The initiation of S1PRM dosing triggers a decrease in heart rate and blood pressure due to initial S1PR1 agonism, which produces the activation of G protein–coupled inwardly rectifying potassium channels followed by myocyte hyperpolarisation and a transitory reduction in excitability; continuous dosing results in the downregulation of S1PR1 as well as a subsequent shift in the S1P receptor profile and an increase in blood pressure (48). Additionally, S1PR3 agonism might play a role in heart rate effects since fingolimod resulted in bradycardia in wild type but not S1PR3 knockout mice (49, 50). However, the possible involvement of S1PR3 in humans remains unclear, and although efforts to develop new modulators with low affinity for S1PR3 such as siponimod and ozanimod have been extensive, minimising S1PR3 binding does not prevent first-dose cardiac effects (9, 12, 14, 51, 52).

### Clinical Considerations

Given the higher incidence of cardiovascular AEs in patients with MS receiving S1PRMs, the following triage of patients might be reasonable. First, clinicians should conduct a comprehensive assessment of patient conditions for the possible risk factors: patients with a history of unstable angina, heart attack, stroke, transient ischaemic attack, decompensated heart failure, class III or IV heart failure, second- or third-degree atrioventricular block, sinoatrial block, or sick sinus syndrome (except for patients with a pacemaker) should avoid their use (11, 38, 44). And electrocardiography is recommended prior to S1PM treatment (41). In addition, multiple sclerosis (MS) commonly affects young adults at the ages 20 to 40 years old, but it can onset at each age. Late-onset multiple sclerosis (LOMS) is defined as symptoms initiating after the age of 50 (53). Comparing with adult-onset multiple sclerosis (AOMS) patients, LOMS patients had significantly more cardiac risk factors, such as heart failure, coronary heart disease and high blood pressure (54, 55), with more combination medications (56). While the incidence of second- or third-degree AV blocks was significantly higher in older patients (≥ 50 years vs. 35–49 years; p = 0.014) after the first dose of fingolimod in a current observational study (57). Therefore, S1PRMs should be carefully selected for LOMS patients. Second, considering the mechanism of S1PRM-induced cardiovascular effects, an up-titration regimen might be necessary to mitigate the heart rate reduction produced by second-generation S1PRMs exhibiting short half-lives (e.g., ozanimod, siponimod, ponesimod), and there exists the potential for inadvertent first-dose effects following relatively brief treatment interruptions, in which cases therapy should be reinitiated with dose titration (58). However, up-titration cannot reduce the incidence of cardiovascular effects with the use of compounds with long half-lives (e.g., fingolimod, cenerimod) because of the less pronounced first-dose-related negative chronotropic effects (5, 58). Third, during S1PRM treatment, especially with fingolimod, heart rates, electrical conduction events, and blood pressure should be monitored *via* active electrocardiography recording for at least 6 h after the first dose and then prolonged according to patient situation (15, 59). In addition to monitoring the cardiovascular AEs occurring after the first dose, long-term follow-up of 1.5–3 years focusing on heart rate and blood pressure is equally important. Finally, a prediction model of cardiovascular risk for patients receiving S1PRMs should be developed since it might help clinicians and pharmacists predict the cardiovascular risks associated with S1PRMs and make individualised medication decisions for each patient. To date, limited observational studies have examined the predictors of fingolimod-induced bradycardia, and parasympathetic function, lower baseline heart rate, body mass index, optic nerve involvement, and baseline heart rate might be independent predictors of a greater heart rate reduction upon the initiation of fingolimod (59).

### Strengths and Limitations

The major strength of this study is that we comprehensively assessed the risk of cardiovascular side effects induced by S1PRMs in patients with MS based on evidence from RCTs. To our knowledge, this is the first meta-analysis to estimate the risk of cardiovascular AEs associated with S1PRMs. Several intrinsic limitations of this study should be recognised. First, due to the limited information retrieved from the included RCTs, this analysis could only compare the incidence and severity of cardiovascular AEs between patients treated with versus without S1PRMs; we were unable to determine the occurrence time, duration, and other details of these AEs. Second, the analyses of individual S1PRMs in our study might be limited by the small number of RCTs of ozanimod and siponimod; therefore, these results must be interpreted cautiously. Third, this study only evaluated the cardiovascular side effects of S1PRMs based on the data from RCTs. And considering a highly selected population in RCTs which usually be younger than 55 years old, there were rarely LOMS patients enrolled in RCTs, whose cardiovascular AEs might be even higher (54, 55, 57, 60). But the proportion of LOMS patients is about 5.01% of all MS cases, which is relatively lower than lower than that of AOMS patients (54, 55); thus, our findings are meaningful to the majority of MS patients. To extend these findings to large patient populations in clinical practice, real-world studies including a wider range of patients, evaluating cardiovascular safety and further meta-analyses assessing the data from real-world studies are necessary.

## Conclusion

In conclusion, by systematically evaluating RCT evidence, this meta-analysis showed that S1PRM use increased the risk for cardiovascular AEs by 1.21 times in MS patients. Compared to non-S1PRM-treated patients, those receiving S1PRMs were at 2.92- and 2.00-fold increased risk for bradyarrhythmia and hypertension, respectively. These findings can help clinicians assess the risk of cardiovascular AEs in patients with MS who are treated with S1PRMs.

## Data Availability Statement

The original contributions presented in the study are included in the article/**Supplementary Material**. Further inquiries can be directed to the corresponding author.

## Author Contributions

Conception and design: C-LM and Z-CG. Administrative support: M-KZ. Provision of study materials or patients: All authors. Collection and assembly of data: ZZ and YL. Data analysis and interpretation: ZZ. Manuscript writing: All authors. All authors contributed to the article and approved the submitted version.

## Funding

This work was supported by the National Science Foundation for Youth Scientists of China [Grant No. 81703613] and the Shanghai “Rising Stars of Medical Talent” Youth Development Program (Youth Medical Talents–Clinical Pharmacist Program). Shanghai Key Clinical Specialty Projects-Clinical Pharmacy to MZ (shslczdzk06502).

## Conflict of Interest

The authors declare that the research was conducted in the absence of any commercial or financial relationships that could be construed as a potential conflict of interest.

## Publisher’s Note

All claims expressed in this article are solely those of the authors and do not necessarily represent those of their affiliated organizations, or those of the publisher, the editors and the reviewers. Any product that may be evaluated in this article, or claim that may be made by its manufacturer, is not guaranteed or endorsed by the publisher.
