# Supplementary material for: Risk for Cardiovascular Adverse Events Associated With Sphingosine-1-Phosphate Receptor Modulators in Patients With Multiple Sclerosis: Insights From a Pooled Analysis of 15 Randomised Controlled Trials
Source: Front Immunol. 2021 Dec 7;12:795574. doi: 10.3389/fimmu.2021.795574 (PMC8688957; doi:10.3389/fimmu.2021.795574)
Supplement: Supplementary file 1 [file DataSheet_1.docx]

***Supplemental File***

**Content**

[Tables 3](#_Toc1776)

[Supplementary Table 1. Search strategy used in Jan 5, 2021 3](#_Toc23584)

[Supplementary Table 2. Patient demographics and clinical characteristics 5](#_Toc28608)

[Supplementary Table 3. Quality assessment 8](#_Toc17857)

[Supplementary Table 4. Sensitivity analysis 10](#_Toc29134)

[Supplementary Table 5. Meta-regression analysis 13](#_Toc361)

[Supplementary Table 6. Subgroup analysis of high-risk cardiovascular adverse events by controls 15](#_Toc32125)

[Supplementary Table 7. Trim and fill method to deal with publication bias 16](#_Toc5635)

[Figures 17](#_Toc15835)

[Supplementary Figure 1. Funnel plot of RCTs with cardiovascular AEs 17](#_Toc27400)

[Supplementary Figure 2. Funnel plot of RCTs with general cardiovascular AEs 18](#_Toc19095)

[Supplementary Figure 3. Funnel plot of RCTs with serious cardiovascular AEs 19](#_Toc14105)

[Supplementary Figure 4. Funnel plot of RCTs with arrhythmia 20](#_Toc20147)

[Supplementary Figure 6. Funnel plot of RCTs with tachyarrhythmia 21](#_Toc13427)

[Supplementary Figure 5. Funnel plot of RCTs with bradyarrhythmia 22](#_Toc29109)

[Supplementary Figure 7. Funnel plot of RCTs with coronary artery disease 23](#_Toc20954)

[Supplementary Figure 8. Funnel plot of RCTs with hypertension 24](#_Toc32607)

[Supplementary Figure 9. Relative risk of cardiovascular AEs 25](#_Toc3046)

[Supplementary Figure 10. Relative risk of cardiovascular AEs by severity 26](#_Toc27196)

[Supplementary Figure 11. Relative risk of arrhythmia 27](#_Toc14825)

[Supplementary Figure 12. Relative risk of bradyarrhythmia 28](#_Toc26500)

[Supplementary Figure 13. Relative risk of tachyarrhythmia 29](#_Toc12990)

[Supplementary Figure 14. Relative risk of hypertension 30](#_Toc5920)

[Supplementary Figure 15. Relative risk of hypotension 31](#_Toc4410)

[Supplementary Figure 16. Relative risk of heart failure 32](#_Toc218)

[Supplementary Figure 17. Relative risk of coronary artery disease 33](#_Toc23843)

[Supplementary Figure 18. Relative risk of acute coronary syndrome 34](#_Toc14331)

[Supplementary Figure 19. Relative risk of acute coronary syndrome 35](#_Toc17617)

[Supplementary Figure 20. Relative risk of bradyarrhythmia by individual S1PRMs 36](#_Toc16608)

[Supplementary Figure 21. Relative risk of bradyarrhythmia associated with fingolimod by dose 37](#_Toc13545)

[Supplementary Figure 22. Relative risk of bradyarrhythmia associated with ozanimod by dose 38](#_Toc28764)

[Supplementary Figure 23. Relative risk of hypertension by individual S1PRMs 39](#_Toc18125)

[Supplementary Figure 24. Relative risk of hypertension associated with fingolimod by dose 40](#_Toc31439)

[Supplementary Figure 25. Relative risk of hypertension associated with ozanimod by dose 41](#_Toc952)

[Supplementary Figure 26. Relative risk of bradyarrhythmia by controls 42](#_Toc22881)

[Supplementary Figure 27. Relative risk of hypertension by controls 43](#_Toc10662)

# **Tables**

**Supplementary Table 1. Search strategy used in Jan 5, 2021**

| **Literature databases** | **Search** | **Query** | | **Items found** |
| --- | --- | --- | --- | --- |
| PUBMED | #1 | All fields | (Multiple Sclerosis) OR (Sclerosis, Multiple) OR (Sclerosis, Disseminated) OR (Disseminated Sclerosis) OR (MS (Multiple Sclerosis)) OR (Multiple Sclerosis, Acute Fulminating) | 92809 |
|  | #2 | All fields | (Fingolimod Hydrochloride) OR FTY-720 OR (FTY 720) OR FTY720 OR Gilenya OR Gilenia OR Fingolimod OR Siponimod OR Mayzent OR BAF312 OR BAF-312 OR ozanimod OR RPC1063 | 3659 |
|  | #3 | All fields | (Randomized Controlled Trial) OR (Clinical Trial) | 1247602 |
|  | #4 | #1 AND #2 AND #3 | | **332** |
| EMBASE | #1 | multiple sclerosis':ti,ab,kw OR 'sclerosis, multiple':ti,ab,kw OR 'sclerosis, disseminated':ti,ab,kw OR 'disseminated sclerosis':ti,ab,kw OR 'multiple sclerosis, acute fulminating':ti,ab,kw | | 123483 |
|  | #2 | fingolimod hydrochloride':ti,ab,kw OR 'fty 720':ti,ab,kw OR fty720:ti,ab,kw OR gilenya:ti,ab,kw OR gilenia:ti,ab,kw OR fingolimod:ti,ab,kw OR siponimod:ti,ab,kw OR mayzent:ti,ab,kw OR baf312:ti,ab,kw OR 'baf 312':ti,ab,kw OR ozanimod:ti,ab,kw OR rpc1063:ti,ab,kw | | 7158 |
|  | #3 | randomized controlled trial':ti,ab,kw OR 'clinical trial':ti,ab,kw | | 334233 |
|  | #4 | #1 AND #2 AND #3 | | **187** |
| COCHRANE | #1 | in Trials (Word variations have been searched) | ("Multiple Sclerosis" OR "Sclerosis, Multiple" OR "Sclerosis, Disseminated" OR "Disseminated Sclerosis" OR "MS (Multiple Sclerosis)" OR "Multiple Sclerosis, Acute Fulminating"):ti,ab,kw (Word variations have been searched) | 10050 |
|  | #2 | in Trials (Word variations have been searched) | ("Fingolimod Hydrochloride" OR FTY-720 OR "FTY 720" OR FTY720 OR Gilenya OR Gilenia OR Fingolimod OR Siponimod OR Mayzent OR BAF312 OR BAF-312 OR ozanimod OR RPC1063):ti,ab,kw (Word variations have been searched) | 787 |
|  | #3 | in Trials (Word variations have been searched) | ("Randomized Controlled Trial" OR "Clinical Trial"):ti,ab,kw (Word variations have been searched) | 674987 |
|  | #4 | #1 AND #2 AND #3 | | **358** |
| Overall |  | | | **867** |

**Supplementary Table 2. Patient demographics and clinical characteristics**

| **Source/Study** | **Interventions/Arms** | **Age (year, mean)** | **Female (%)** | **EDSS scores (mean)** | **Disease duration (year, mean)** | **R in 1 (mean)** | **R in 2 (mean)** | **N of p without G+ (%)** | **N of G+ (mean)** | **V of lesions (mm3, mean)** | **Brain volume (cm3, mean)** |
| --- | --- | --- | --- | --- | --- | --- | --- | --- | --- | --- | --- |
| Kappos et al, 2006 ( FTY720 D2201) | fingolimod 1.25 mg/d | 38.0 (9.54) | 75 | 8.6 (0.3-50.2) | 1.3 (0-5) | 1.9 (0-8) | 42 | 2.8 | 9515.8 | NR | NR |
|  | fingolimod 5.0 mg/d | 38.3 (10.51) | 71 | 9.5 (0.5-42.2) | 1.3 (0-4) | 1.9 (0-8) | NR | NR | NR | NR | NR |
|  | placebo | 37.0 (8.80) | 66 | 8.4 (0.2-28.2) | 1.2 (0-5) | 1.8 (0-6) | NR | NR | NR | NR | NR |
| Cohen et al, 2010 (TRANSFORMS) | fingolimod 1.25 mg/d | 35.8 (8.4) | 68.8 | 7.3 (6.0) | 1.5 (0.9) | 2.2 (1.2) | 63.9 | 1.17 | 5059 | 1525.7 | 62.3 |
|  | fingolimod 0.5 mg/d | 36.7 (8.8) | 65.4 | 7.5 (6.2) | 1.5 (1.2) | 2.3 (2.2) | NR | NR | NR | NR | NR |
|  | interferon beta-1a 30 μg/w | 36.0 (8.3) | 67.8 | 7.4 (6.3) | 1.5 (0.8) | 2.3 (1.2) | NR | NR | NR | NR | NR |
| Kappos et al, 2010 (FREEDOMS) | fingolimod 1.25 mg/d | 37.4 (8.9) | 68.8 | 8.4 (6.9) | 1.5 (0.8) | 2.1 (1.3) | 61.5 | 1.6 | 6376 | 1514 | 40.9 |
|  | fingolimod 0.5 mg/d | 36.6 (8.8) | 69.6 | 8.0 (6.6) | 1.5 (0.8) | 2.1 (1.1) | NR | NR | NR | NR | NR |
|  | placebo | 37.2 (8.6) | 71.3 | 8.1 (6.4) | 1.4 (0.7) | 2.2 (1.2) | NR | NR | NR | NR | NR |
| Saida et al, 2012 | fingolimod 1.25 mg/d | 36.0 (9.3) | 68.4 | 7.1 (5.3) | 1.5 (0.9) | 2.3 (1.7) | 55.6 | 1.4 | NR | NR | NR |
|  | fingolimod 0.5 mg/d | 35.0 (9.0) | 70.2 | 8.2 (6.8) | 1.4 (1.0) | 2.2 (1.4) | NR | NR | NR | NR | NR |
|  | placebo | 35.0 (8.9) | 68.4 | 8.2 (7.3) | 1.7 (1.6) | 2.8 (3.0) | NR | NR | NR | NR | NR |
| Selmaj et al, 2013 (BOLD) | siponimod 10 mg/d | 36.4 (8.4) | 60 | 6.0 (6.1) | 1.4 (0.8) | 2.0 (1.0) | 49.2 | 2.0 | NR | NR | NR |
|  | siponimod 2 mg/d | 37.4 (8.9) | 69 | 7.2 (6.8) | 1.3 (0.6) | 2.1 (1.0) | NR | NR | NR | NR | NR |
|  | siponimod 1.25 mg/d | 35.4 (8.9) | 74 | 7.2 (6.6) | 1.3 (0.6) | 1.8 (0.8) | NR | NR | NR | NR | NR |
|  | siponimod 0.5 mg/d | 36.0 (8.8) | 70 | 8.7 (7.3) | 1.5 (0.9) | 1.8 (1.0) | NR | NR | NR | NR | NR |
|  | siponimod 0.25 mg/d | 37.4 (8.4) | 82 | 7.7 (5.7) | 1.4 (0.8) | 2.0 (0.9) | NR | NR | NR | NR | NR |
|  | placebo | 35.4 (8.6) | 73 | 8.0 (6.6) | 1.3 (0.6) | 1.8 (0.7) | NR | NR | NR | NR | NR |
| Calabresi et al, 2014 (FREEDOMS II) | fingolimod 1.25 mg/d | 40.9 (8.9) | 76 | 10.8 (8.2) | 1.5 (1.0) | 2.3 (2.0) | 64.4 | 1.3 | 5319 | 1521 | 74.8 |
|  | fingolimod 0.5 mg/d | 40.6 (8.4) | 77 | 10.4 (8.0) | 1.4 (0.9) | 2.2 (1.4) | NR | NR | NR | NR | NR |
|  | placebo | 40.1 (8.4) | 81 | 10.6 (7.9) | 1.5 (0.9) | 2.2 (1.5) | NR | NR | NR | NR | NR |
| Fox et al, 2014 (EPOC) | fingolimod 0.5 mg/d | 46.0 (9.82) | 76.1 | 12.1 (8.38) | 0.8 (1.20) | 1.4 (2.04) | NR | NR | NR | NR | 100.0 |
|  | iDMT (interferon beta or glatirameracetate) | 45.1 (9.82) | 79.1 | 11.7 (8.44) | 0.8 (1.32) | 1.4 (1.93) | NR | NR | NR | NR | NR |
| Cohen et al, 2016 (RADIANCE) | ozanimod 0.5 mg/d | 38.1 (9.2) | 69 | 6.0 (6.4) | 1.5 (1.2) | 2.0 (1.8) | 53.1 | 1.2 | NR | NR | NR |
|  | ozanimod 1.0 mg/d | 38.4 (9.8) | 71 | 6.2 (5.8) | 1.3 (0.7) | 1.9 (1.1) | NR | NR | NR | NR | NR |
|  | placebo | 39.0 (8.7) | 70 | 8.1 (7.0) | 1.3 (0.6) | 1.8 (1.0) | NR | NR | NR | NR | NR |
| Lublin et al, 2016 (INFORMS) | fingolimod 1.25 mg/d | 47.8 (8.47) | 48.3 | NR | NR | NR | NR | NR | NR | NR | NR |
|  | fingolimod 0.5 mg/d | 48.5 (8.6) | 49 | 5.8 (2.5) | 0 | 0 | NR | NR | 9794.5 | 1491.4 | 78 |
|  | placebo | 48.5 (8.3) | 48 | 5.9 (2.4) | 0 | 0 |  |  |  |  |  |
| Popova et al, 2017 (GIMN) | fingolimod 0.5 mg/d | 35.4 (2.33） | 70.4 | 4.71 | NR | 2.2 (0.13) | NR | NR | NR | NR | NR |
|  | interferon beta-1a 44 μg or glatirameracetate | 36.4 (4.41） | 73.5 | 10.66 | NR | 2.0 (0.24) | NR | NR | NR | NR | NR |
| Comi et al, 2017 (GOLDEN) | fingolimod 0.5 mg/d | 35.2 (9.22) | 71.25 | 4.97 (6.67) | 1.45 (0.79) | 1.90 (0.84) | NR | 0.79 | 9956.5 | 1401.9 | 49.1 |
|  | interferon beta-1b 250 µg every other day | 32.89 (8.10) | 67.86 | 4.71 (6.47） | 1.18 (0.48) | 1.54 (0.84) | NR | NR | NR | NR | NR |
| Kappos et al, 2018 (EXPAND) | siponimod 2 mg/d | 48.0 (7.8) | 61 | 17.1 (8.4) | 0.2 (0.5) | 0.7 (1.2) | 75.6 | NR | 11016.6 | 1423 | 78.3 |
|  | placebo | 48.1 (7.9) | 59 | 16.1 (8.2) | 0.3 (0.6) | 0.7 (1.2) | NR | NR | NR | NR | NR |
| Cree et al, 2018 (PREFERMS) | fingolimod 0.5 mg/d | 41.5 (10.84) | 71.3 | 7.29 (8.21) | 0.6 (0.95) | 0.9 (1.51) | NR | 0.97 | 7545.0 | 1516.4 | NR |
|  | iDMT (interferon beta or glatirameracetate) | 41.9 (10.39) | 74.9 | 7.21 (7.66) | 0.6 (0.94) | 0.9 (1.41) | NR | NR | NR | NR | NR |
| Cohen et al, 2019 (RADIANCE) | ozanimod 0.5 mg/d | 35.4 (8.8) | 65.4 | 6.2 (5.55) | 1.4 (0.64) | 1.8 (0.90) | NR | 1.8 | 11439 | 1448.2 | 28.9 |
|  | ozanimod 1.0 mg/d | 36.0 (8.9) | 67.2 | 6.9 (6.20) | 1.3 (0.56) | 1.7 (0.82) | NR | NR | NR | NR | NR |
|  | interferon beta-1a 30µg/w | 35.1 (9.1) | 68.9 | 6.4 (6.07) | 1.3 (0.58) | 1.8 (0.86） | NR | NR | NR | NR | NR |
| Comi et al, 2019 (SUNBEAM) | ozanimod 0.5 mg/d | 36.0 (9.4) | 69.0 | 7.2 (6.3) | 1.3 (0.6) | 1.7 (0.8) | NR | 1.7 | 13067 | 1448.9 | 30.5 |
|  | ozanimod 1.0 mg/d | 34.8 (9.2) | 63.3 | 6.9 (6.4) | 1.3 (0.6) | 1.8 (0.9) | NR | NR | NR | NR | NR |
|  | interferon beta-1a 30µg/w | 35.9 (9.1) | 67.0 | 6.9 (5.9) | 1.3 (0.6) | 1.7 (0.8) | NR | NR | NR | NR | NR |
| Butzkueven et al, 2020 (REVEAL) | fingolimod 0.5 mg/d | 38.2 (8.8) | 68.5 | 8.1 (7.7) | 1.9 (0.6) | NR | NR | 2.45 | 1140 | NR | 50.0 |
|  | Natalizumab 300 mg/4w | 34.9 (8.7) | 70.4 | 6.8 (7.0) | 1.9 (0.7) | NR | NR | NR | NR | NR | NR |
| Cree et al, 2020 (ASSESS) | fingolimod 0.5 mg/d | 40.3 (11.1) | 75.0 | 4.3 (5.9) | 1.4 (0.8) | 2.2 (1.6) | 64.0 | 1.8 | 9800 | 1508.2 | 53.1 |
|  | fingolimod 0.25 mg/d | 38.9 (11.0) | 74.6 | 4.6 (6.4) | 1.3 (0.7) | 2.1 (1.3) | NR | NR | NR | NR | NR |
|  | glatirameracetate 20 mg/d s.c. | 39.6 (10.8) | 74.4 | 4.7 (6.2) | 1.4 (0.8) | 2.2 (1.5) | NR | NR | NR | NR | NR |

EDSS: expanded disability status scale; NR: not reported; SD: Standard Deviation; R in 1, Relapse in previous 1 year; R in 2, Relapse in previous 2 year; N of p without G+, Number of patients without G+ lesions on T1-weighted images; N of G+, Number of G+ lesions on T1-weighted images; V of lesions, Volume of lesions on T2-weighted images; Brain volume, Normalized brain volume; N of previous treatment, Number of patients given previous treatment

**Supplementary Table 3. Quality assessment**

| Study | Random sequence generation | Allocation concealment | Blinding of participants and personnel | Blinding of outcome assessment | Incomplete outcome data | Selective reporting | Other bias |
| --- | --- | --- | --- | --- | --- | --- | --- |
| Kappos et al, 2006 | Low | Low | Low | Low | Low | Low | Unclear |
| Cohen et al, 2010 | Low | Low | Low | Low | Unclear | Low | Unclear |
| Kappos et al, 2010 | Low | Low | Low | Low | Low | Low | Unclear |
| Saida et al, 2012 | Unclear | Low | Low | Low | Low | Low | Unclear |
| Selmaj et al, 2013 | Low | Low | Low | Low | Low | Low | Unclear |
| Calabresi et al, 2014 | Low | Low | Low | Low | Low | Low | Unclear |
| Fox et al, 2014 | Low | High | High | High | High | Low | Unclear |
| Cohen et al, 2016 | Low | Low | Low | Low | Low | Low | Unclear |
| Lublin et al, 2016 | Low | Low | Low | Low | Low | Low | Unclear |
| Popova et al, 2017 | Unclear | High | High | High | Unclear | Low | Unclear |
| Comi et al, 2017 | Unclear | High | High | High | Low | Unclear | Unclear |
| Kappos et al, 2018 | Low | Low | Low | Low | Low | Low | Unclear |
| Cree et al, 2018 | Low | High | High | High | Low | Low | Unclear |
| Cohen et al, 2019 | Low | Low | Low | Low | Low | Low | Unclear |
| Comi et al, 2019 | Low | Low | Low | Low | Low | Low | Unclear |
| Butzkueven et al, 2020 | Unclear | High | High | Unclear | High | Low | Unclear |
| Cree et al, 2020 | Low | High | High | Low | Low | Low | Unclear |

Low: low risk; unclear: unclear risk; High: high risk.

**Supplementary Table 4. Sensitivity analysis**

| **Omitted RCTs** | **RR (95%CI)** | **Omitted RCTs** | **RR (95%CI)** |
| --- | --- | --- | --- |
| **Cardiovascular AEs** | | **General cardiovascular AEs** | |
| Butzkueven et al, 2020 (REVEAL) | 2.21 (1.57-3.11) | Calabresi et al, 2014 (FREEDOMS II) | 2.18 (1.57-3.02) |
| Calabresi et al, 2014 (FREEDOMS II) | 2.1 (1.48-2.98) | Cohen et al, 2010 (TRANSFORMS) | 2.31 (1.64-3.25) |
| Cohen et al, 2010 (TRANSFORMS) | 2.12 (1.49-3.01) | Cohen et al, 2016 (RADIANCE) | 2.5 (1.84-3.41) |
| Cohen et al, 2016 (RADIANCE) | 2.4 (1.71-3.36) | Cohen et al, 2019 (RADIANCE) | 2.39 (1.7-3.38) |
| Cohen et al, 2019 (RADIANCE) | 2.29 (1.59-3.3) | Comi et al, 2017 (GOLDEN) | 2.27 (1.63-3.18) |
| Comi et al, 2017 (GOLDEN) | 2.22 (1.58-3.14) | Comi et al, 2019 (SUNBEAM) | 2.19 (1.57-3.04) |
| Comi et al, 2019 (SUNBEAM) | 2.16 (1.52-3.07) | Cree et al, 2020 (ASSESS) | 2.3 (1.66-3.18) |
| Cree et al, 2020 (ASSESS) | 2.17 (1.51-3.12) | Cree et al, 2018 (PREFERMS) | 2.03 (1.56-2.66) |
| Cree et al, 2018 (PREFERMS) | 2.18 (1.54-3.07) | Fox et al, 2014 (EPOC) | 2.46 (1.76-3.42) |
| Fox et al, 2014 (EPOC) | 1.94 (1.44-2.62) | Kappos et al, 2006 (FTY720 D2201) | 2.35 (1.65-3.35) |
| Kappos et al, 2006 (FTY720 D2201) | 2.35 (1.64-3.35) | Kappos et al, 2010 (FREEDOMS) | 2.41 (1.66-3.49) |
| Kappos et al, 2010 (FREEDOMS) | 2.43 (1.76-3.36) | Kappos et al, 2018 (EXPAND) | 2.38 (1.65-3.44) |
| Kappos et al, 2018 (EXPAND) | 2.3 (1.55-3.42) | Lublin et al, 2016 (INFORMS) | 2.25 (1.64-3.1) |
| Lublin et al, 2016 (INFORMS) | 2.28 (1.54-3.37) | Saida et al, 2012 | 2.33 (1.66-3.26) |
| Popova, E.V et al, 2017 (GIMN) | 2.22 (1.58-3.14) | Selmaj et al, 2013 (BOLD) | 2.18 (1.57-3.02) |
| Saida et al, 2012 | 2.14 (1.52-3.01) | **Arrhythmia** | |
| Selmaj et al, 2013 (BOLD) | 2.19 (1.53-3.12) | Butzkueven et al, 2020 (REVEAL) | 2.83 (1.86-4.32) |
| **Serious AEs** | | Calabresi et al, 2014 (FREEDOMS II) | 2.68 (1.7-4.23) |
| Butzkueven et al, 2020 (REVEAL) | 1.75 (1.07-2.86) | Cohen et al, 2010 (TRANSFORMS) | 2.79 (1.83-4.24) |
| Calabresi et al, 2014 (FREEDOMS II) | 1.64 (0.99-2.72) | Cohen et al, 2016 (RADIANCE) | 3.06 (2.04-4.59) |
| Cohen et al, 2010 (TRANSFORMS) | 1.46 (0.97-2.21) | Cohen et al, 2019 (RADIANCE) | 2.76 (1.82-4.18) |
| Cohen et al, 2019 (RADIANCE) | 1.8 (1.08-3) | Comi et al, 2017 (GOLDEN) | 2.93 (1.94-4.4) |
| Comi et al, 2017 (GOLDEN) | 1.78 (1.09-2.92) | Comi et al, 2019 (SUNBEAM) | 2.81 (1.84-4.29) |
| Comi et al, 2019 (SUNBEAM) | 1.72 (1.05-2.8) | Cree et al, 2020 (ASSESS) | 2.56 (1.74-3.78) |
| Cree et al, 2020 (ASSESS) | 1.9 (1.18-3.06) | Cree et al, 2018 (PREFERMS) | 2.79 (1.83-4.24) |
| Cree et al, 2018 (PREFERMS) | 1.7 (1.04-2.77) | Fox et al, 2014 (EPOC) | 2.54 (1.74-3.7) |
| Fox et al, 2014 (EPOC) | 1.67 (1.04-2.68) | Kappos et al, 2006 ( FTY720 D2201) | 3.08 (2.07-4.59) |
| Kappos et al, 2006 (FTY720 D2201) | 1.85 (1.1-3.1) | Kappos et al, 2010 (FREEDOMS) | 2.98 (1.89-4.68) |
| Kappos et al, 2010 (FREEDOMS) | 1.99 (1.33-2.97) | Kappos et al, 2018 (EXPAND) | 2.91 (1.79-4.74) |
| Kappos et al, 2018 (EXPAND) | 1.82 (1.07-3.11) | Lublin et al, 2016 (INFORMS) | 3.03 (1.96-4.69) |
| Lublin et al, 2016 (INFORMS) | 1.81 (1.05-3.1) | Popova, E.V et al, 2017 (GIMN) | 2.87 (1.88-4.37) |
| Popova, E.V et al, 2017 (GIMN) | 1.78 (1.08-2.92) | Saida et al, 2012 | 2.72 (1.81-4.09) |
| Saida et al, 2012 | 1.78 (1.08-2.94) | Selmaj et al, 2013 (BOLD) | 2.86 (1.82-4.51) |
| Selmaj et al, 2013 (BOLD) | 1.67 (1.04-2.69) | **Tachyarrhythmia** | |
| **Bradyarrhythmia** | | Calabresi et al, 2014 (FREEDOMS II) | 1.45 (0.61-3.43) |
| Butzkueven et al, 2020 (REVEAL) | 2.94 (1.89-4.57) | Cohen et al, 2010 (TRANSFORMS) | 1.44 (0.61-3.39) |
| Calabresi et al, 2014 (FREEDOMS II） | 2.78 (1.7-4.54) | Cohen et al, 2016 (RADIANCE) | 1.61 (0.66-3.9) |
| Cohen et al, 2010 (TRANSFORMS) | 2.78 (1.85-4.17) | Cohen et al, 2019 (RADIANCE) | 1.39 (0.6-3.2) |
| Cohen et al, 2016 (RADIANCE) | 3.15 (2.11-4.68) | Comi et al, 2019 (SUNBEAM) | 1.5 (0.63-3.59) |
| Cohen et al, 2019 (RADIANCE) | 2.94 (1.89-4.57) | Cree et al, 2020 (ASSESS) | 1.78 (0.79-4.01) |
| Comi et al, 2017 (GOLDEN) | 3.03 (1.99-4.61) | Fox et al, 2014 (EPOC) | 1.14 (0.52-2.51) |
| Comi et al, 2019 (SUNBEAM) | 2.95 (1.89-4.59) | Kappos et al, 2006 ( FTY720 D2201) | 2.18 (1.06-4.49) |
| Cree et al, 2020 (ASSESS) | 2.74 (1.88-3.99) | Kappos et al, 2010 (FREEDOMS) | 1.87 (0.83-4.18) |
| Cree et al, 2018 (PREFERMS) | 2.88 (1.86-4.47) | Kappos et al, 2018 (EXPAND) | 1.61 (0.67-3.9) |
| Fox et al, 2014 (EPOC) | 2.76 (1.85-4.11) | Lublin et al, 2016 (INFORMS) | 1.44 (0.61-3.4) |
| Kappos et al, 2006 (FTY720 D2201) | 2.86 (1.85-4.42) | Popova, E.V et al, 2017 (GIMN) | 1.6 (0.67-3.8) |
| Kappos et al, 2010 (FREEDOMS) | 3.02 (1.87-4.9) | Selmaj et al, 2013 (BOLD) | 1.48 (0.61-3.6) |
| Kappos et al, 2018 (EXPAND) | 3.05 (1.8-5.16) | **Coronary artery disease** | |
| Lublin et al, 2016 (INFORMS) | 3.25 (2.14-4.93) | Calabresi et al,2014（FREEDOMS II） | 0.76 (0.36-1.58) |
| Popova, E.V et al, 2017 (GIMN) | 2.99 (1.94-4.62) | Cohen et al,2010(TRANSFORMS) | 0.74 (0.36-1.51) |
| **Hypertension** | | Cohen et al,2016 (RADIANCE) | 0.7 (0.34-1.45) |
| Calabresi et al, 2014 (FREEDOMS II) | 1.85 (1.39-2.45) | Cohen et al,2019 (RADIANCE) | 0.7 (0.34-1.45) |
| Cohen et al, 2010 (TRANSFORMS) | 1.97 (1.45-2.7) | Cree et al, 2020(ASSESS) | 0.71 (0.34-1.48) |
| Cohen et al, 2016 (RADIANCE) | 2.02 (1.49-2.74) | Cree et al,2018(PREFERMS) | 0.61 (0.3-1.26) |
| Cohen et al, 2019 (RADIANCE) | 2.09 (1.51-2.88) | Kappos et al,2006 ( FTY720 D2201) | 0.74 (0.36-1.52) |
| Comi et al, 2019 (SUNBEAM) | 1.95 (1.44-2.64) | Kappos et al,2010(FREEDOMS) | 0.72 (0.34-1.53) |
| Cree et al, 2020 (ASSESS) | 2.01 (1.46-2.77) | Kappos et al,2018 (EXPAND) | 0.65 (0.29-1.45) |
| Fox et al, 2014 (EPOC) | 1.72 (1.41-2.11) | Lublin et al,2016(INFORMS) | 0.55 (0.25-1.19) |
| Kappos et al, 2006 (FTY720 D2201) | 2.08 (1.51-2.86) | **Chronic coronary syndrome** | |
| Kappos et al, 2010 (FREEDOMS) | 2.07 (1.49-2.87) | Calabresi et al, 2014 (FREEDOMS II) | 1 (1.37-2.72) |
| Kappos et al, 2018 (EXPAND) | 2.14 (1.54-2.96) | Cohen et al, 2016 (RADIANCE) | 0.86 (0.32-2.29) |
| Lublin et al, 2016 (INFORMS) | 2.13 (1.54-2.95) | Cohen et al, 2019 (RADIANCE) | 0.72 (0.28-1.89) |
| **Hypotension** | | Cree et al, 2020 (ASSESS) | 0.8 (0.3-2.12) |
| Cohen et al, 2016 (RADIANCE) | 0.91 (0.34-2.45) | Kappos et al, 2006 ( FTY720 D2201) | 0.88 (0.34-2.31) |
| Cree et al, 2020 (ASSESS) | 1.02 (0.47-2.23) | Kappos et al, 2010 (FREEDOMS) | 0.73 (0.28-1.91) |
| Fox et al, 2014 (EPOC) | 0.9 (0.41-1.95) | Kappos et al, 2018 (EXPAND) | 0.79 (0.29-2.13) |
| Kappos et al, 2018 (EXPAND) | 0.93 (0.43-2.04) | Lublin et al, 2016 (INFORMS) | 0.76 (0.27-2.18) |
| Lublin et al, 2016 (INFORMS) | 1.26 (0.51-3.13) | **Acute coronary syndrome** | |
| Saida et al, 2012 | 0.89 (0.41-1.95) | Cohen et al, 2010 (TRANSFORMS) | 0.58 (0.2-1.69) |
| **Heart failure** | | Cohen et al, 2019 (RADIANCE) | 0.58 (0.2-1.69) |
| Calabresi et al, 2014 (FREEDOMS II） | 1.14 (0.18-7.34) | Cree et al, 2020 (ASSESS) | 0.54 (0.18-1.65) |
| Kappos et al, 2006 (FTY720 D2201) | 1.14 (0.18-7.34) | Cree et al, 2018 (PREFERMS) | 0.39 (0.13-1.13) |
| Kappos et al, 2010 (FREEDOMS) | 1.15 (0.18-7.38) | Kappos et al, 2006 (FTY720 D2201) | 0.54 (0.18-1.64) |
| Saida et al, 2012 | 2.96 (0.47-18.75) | Kappos et al, 2010 (FREEDOMS) | 0.63 (0.22-1.84) |
|  |  | Kappos et al, 2018 (EXPAND) | 0.52 (0.15-1.77) |
|  |  | Lublin et al, 2016 (INFORMS) | 0.38 (0.13-1.12) |

RR: relative risk; CI: confidence interval; RCT, randomized clinical trial; AEs, adverse events.

**Supplementary Table 5. Meta-regression analysis**

| **Outcomes**  **P value**  **Variable** | **Age (year, mean)** | **Female (%)** | **EDSS scores (mean)** | **Disease duration (year, mean)** | **R in 1 (mean)** | **R in 2 (mean)** | **N of p without G+ (%)** | **N of G+ (mean)** | **V of lesions (mm3, mean)** | **Brain volume (cm3, mean)** | **N of previous treatment (%)** |
| --- | --- | --- | --- | --- | --- | --- | --- | --- | --- | --- | --- |
| **Overall cardiovascular AEs** | 0.556 | 0.605 | 0.683 | 0.740 | 0.949 | 0.993 | 0.678 | 0.992 | 0.895 | 0.793 | 0.953 |
| **General cardiovascular AEs** | 0.303 | 0.483 | 0.284 | 0.451 | 0.406 | 0.427 | 0.405 | 0.988 | 0.812 | 0.591 | 0.579 |
| **Serious cardiovascular AEs** | 0.556 | 0.605 | 0.683 | 0.740 | 0.949 | 0.993 | 0.678 | 0.992 | 0.895 | 0.793 | 0.953 |
| **Arrhythmia** | 0.354 | 0.818 | 0.418 | 0.255 | 0.533 | 0.595 | 0.451 | 0.821 | 0.996 | 0.867 | 0.475 |
| **Bradyarrhythmia** | 0.295 | 0.220 | 0.388 | 0.212 | 0.506 | 0.576 | 0.384 | 0.588 | 0.984 | 0.870 | 0.371 |
| **Tachyarrhythmia** | 0.687 | 0.675 | 0.861 | 0.637 | 0.692 | 0.674 | 0.849 | 0.819 | 0.998 | 0.959 | 0.570 |
| **Hypertension** | 0.482 | 0.536 | 0.410 | 0.598 | 0.492 | 0.506 | 0.567 | 0.902 | 0.786 | 0.655 | 0.734 |
| **Coronary artery disease** | 0.517 | 0.605 | 0.491 | 0.632 | 0.614 | 0.641 | 0.578 | 0.999 | 0.699 | 0.651 | 0.708 |
| **Acute coronary syndrome** | 0.708 | 0.840 | 0.668 | 0.637 | 0.668 | 0.691 | 0.734 | 0.979 | 0.804 | 0.694 | 0.756 |
| **Chronic coronary syndrome** | 0.692 | 0.755 | 0.753 | 0.967 | 0.906 | 0.923 | 0.848 | 0.871 | 0.998 | 0.990 | 0.725 |
| **Heart failure** | 0.932 | 0.930 | 0.895 | 0.831 | 0.874 | 0.858 | 0.791 | 0.586 | 0.744 | 0.997 | 0.755 |
| **Hypotension** | 0.960 | 0.721 | 0.897 | 0.574 | 0.614 | 0.808 | 0.547 | 0.614 | 0.732 | 0.806 | 0.980 |
| **Vascular disorders** | 0.434 | 0.383 | 0.492 | 0.586 | 0.986 | 0.980 | 0.935 | 0.957 | 0.606 | 0.879 | 0.553 |
| **Venous disorder** | 0.520 | 0.763 | 0.479 | 0.844 | 0.553 | 0.647 | 0.795 | 0.720 | 0.667 | 0.816 | 0.602 |
| **Arterial disorder** | 0.888 | 0.798 | 0.669 | 0.758 | 0.226 | 0.963 | 0.985 | 0.969 | 0.700 | 0.972 | 0.922 |
| **Haemorrhage** | 0.917 | 0.890 | 0.626 | 0.675 | 0.499 | 0.568 | 0.929 | 0.853 | 0.710 | 0.966 | 0.979 |

R in 1, Relapse in previous 1 year; R in 2, Relapse in previous 2 year; N of p without G+, Number of patients without G+ lesions on T1-weighted images; N of G+, Number of G+ lesions on T1-weighted images; V of lesions, Volume of lesions on T2-weighted images; Brain volume, Normalized brain volume; N of previous treatment, Number of patients given previous treatment; AEs, adverse events.

**Supplementary Table 6. Subgroup analysis of high-risk cardiovascular adverse events by controls**

|  | **Subgroup** | **No.s** | **S1PRMs case/No.** | **Controls case/No.** | **RR (95% Cl)** | **I^2^ (%)** | **P**interaction |
| --- | --- | --- | --- | --- | --- | --- | --- |
| **Bradyarrhythmia** |  |  |  |  |  |  |  |
|  | Other DMTs | 9 | 80/4138 | 1/3020 | **5.62 (1.82, 17.32)** | 23.3 | 0.445 |
|  | Placebo | 8 | 288/3735 | 53/2105 | **2.57 (1.71, 3.87)** | 34.3 |  |
| **Hypertension** |  |  |  |  |  |  |  |
|  | Other DMTs | 5 | 181/3751 | 44/2423 | **2.71 (1.52, 4.85)** | 65.1 | 0.225 |
|  | Placebo | 6 | 340/3764 | 112/1987 | **1.64 (1.25, 2.17)** | 34.3 |  |

I^2^, heterogeneity; No. s, numbers of studies; RR, relative risk; 95% CI, 95% confidence interval; S1PRMs, sphingosine-1-phosphate receptor modulators; DMTs, disease modulating therapies.

**Supplementary Table 7. Trim and fill method to deal with publication bias**

| **Outcomes** | **Publication bias** | **Before trim and fill** | | **After trim and fill** | |
| --- | --- | --- | --- | --- | --- |
|  | **P for Egger’s test** | **No. of studies** | **RR (95% Cl)** | **No. of studies** | **RR (95% Cl)** |
| General cardiovascular AEs | 0.011 | 14 | 2.30 (1.67, 3.17) | 18 | 1.849 (1.309, 2.611) |
| arrhythmia | 0.010 | 17 | 2.83 (1.88, 4.26) | 20 | 2.240 (1.427, 3.516) |
| bradyarrhythmia | 0.061 | 17 | 2.92 (1.91, 4.46) | 20 | 2.461 (1.518, 3.990) |
| hypertension | 0.014 | 11 | 2.00 (1.49, 2.67) | 14 | 1.581 (1.122, 2.229) |

AEs, adverse events;RR, relative risk; 95% CI, 95% confidence interval.

# **
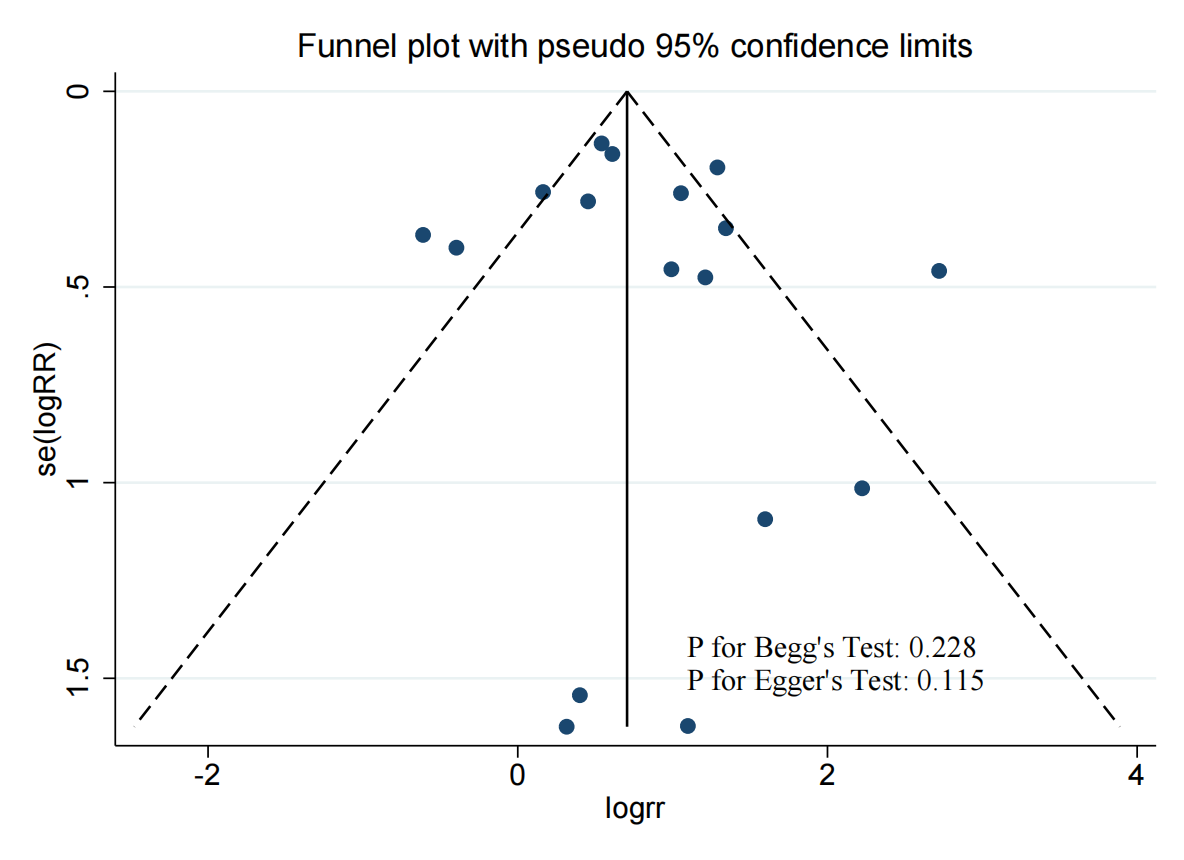
Figures**

# Supplementary Figure 1. Funnel plot of RCTs with cardiovascular AEs

**Supplementary Figure 2. Funnel plot of RCTs with general cardiovascular AEs
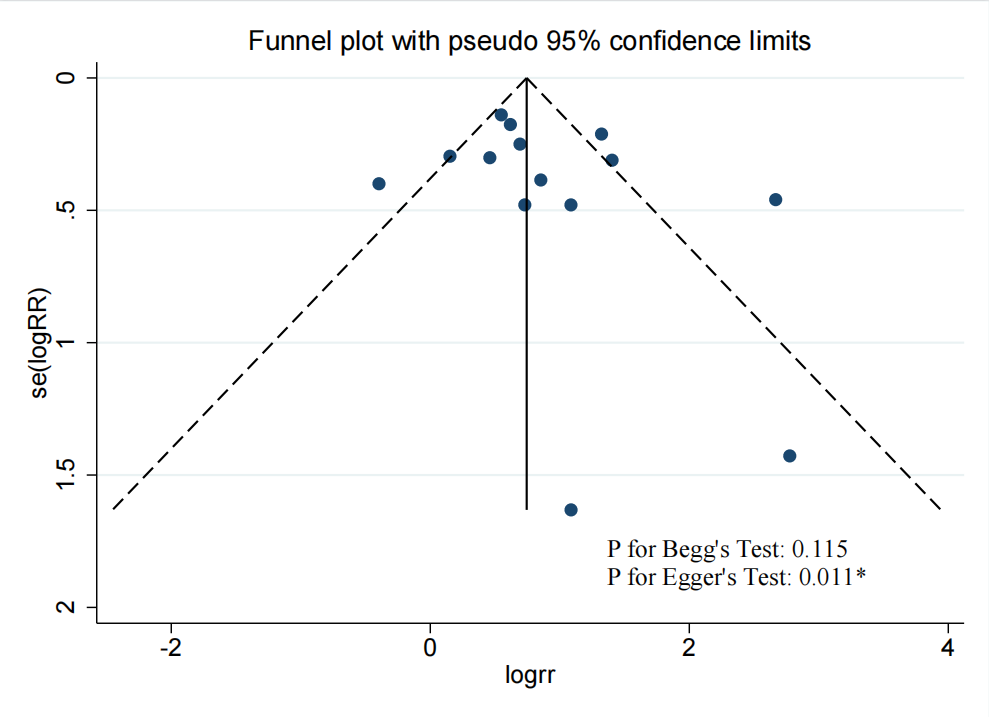
**


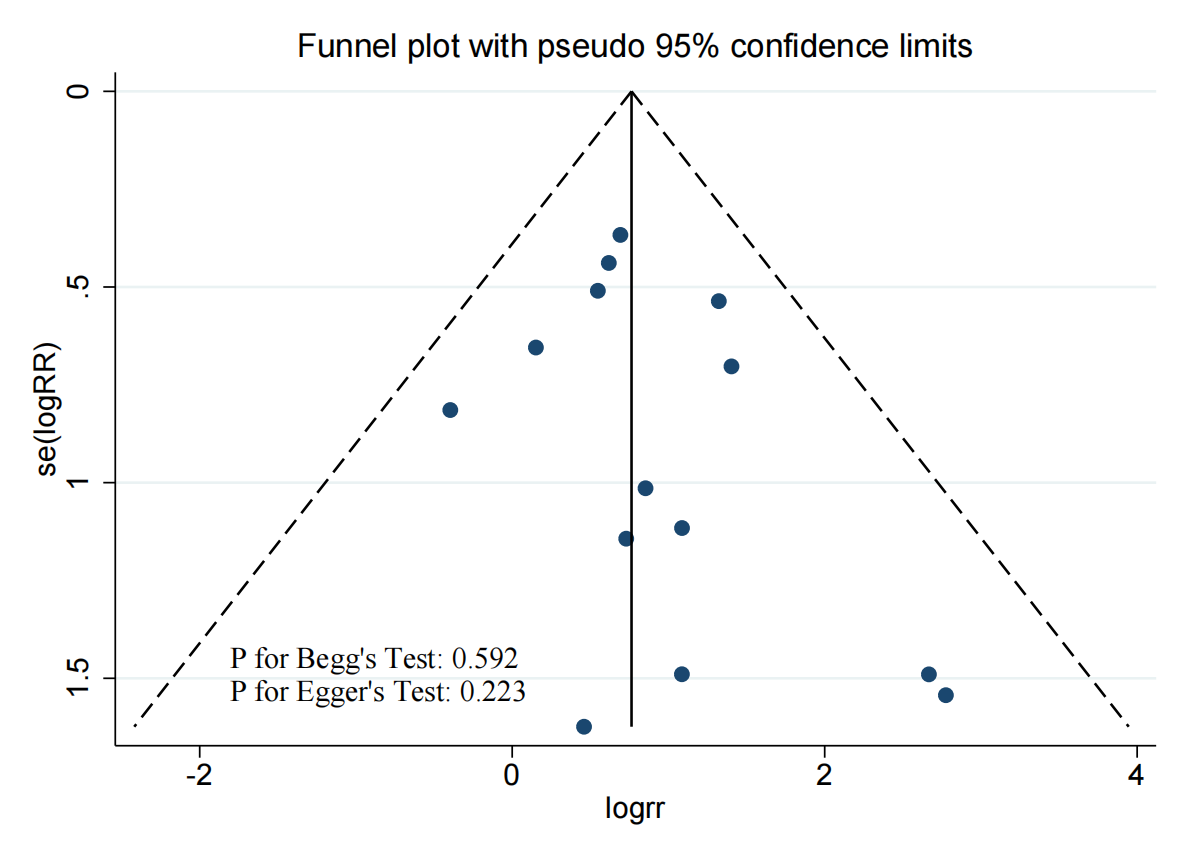
**Supplementary Figure 3. Funnel plot of RCTs with serious cardiovascular AEs**


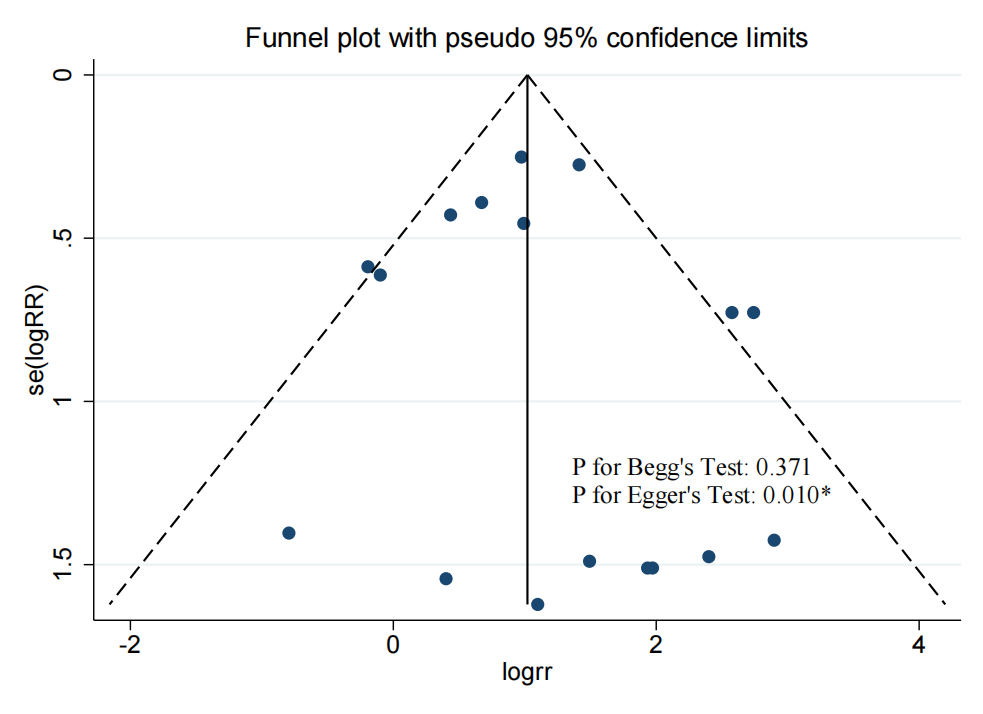
**Supplementary Figure 4. Funnel plot of RCTs with arrhythmia**

**Supplementary Figure 6. Funnel plot of RCTs with tachyarrhythmia**
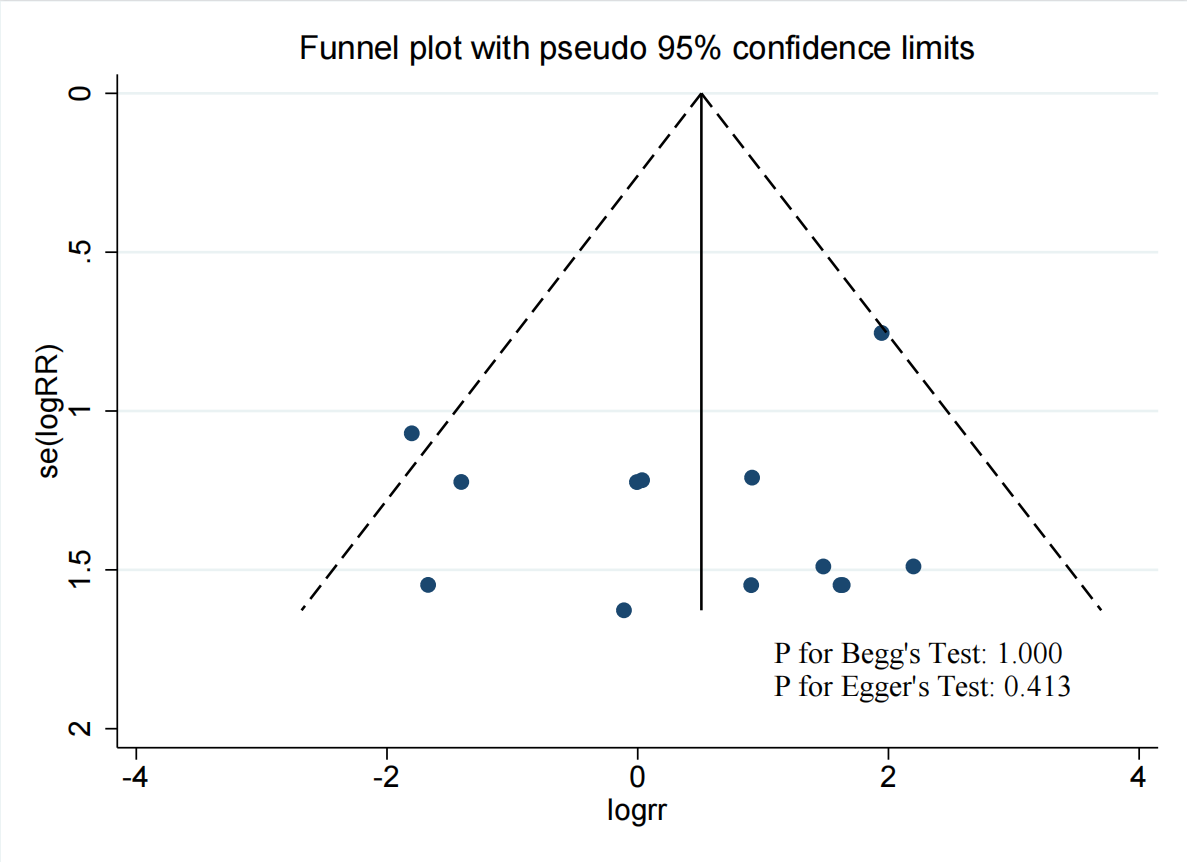


**Supplementary Figure 5. Funnel plot of RCTs with bradyarrhythmia**
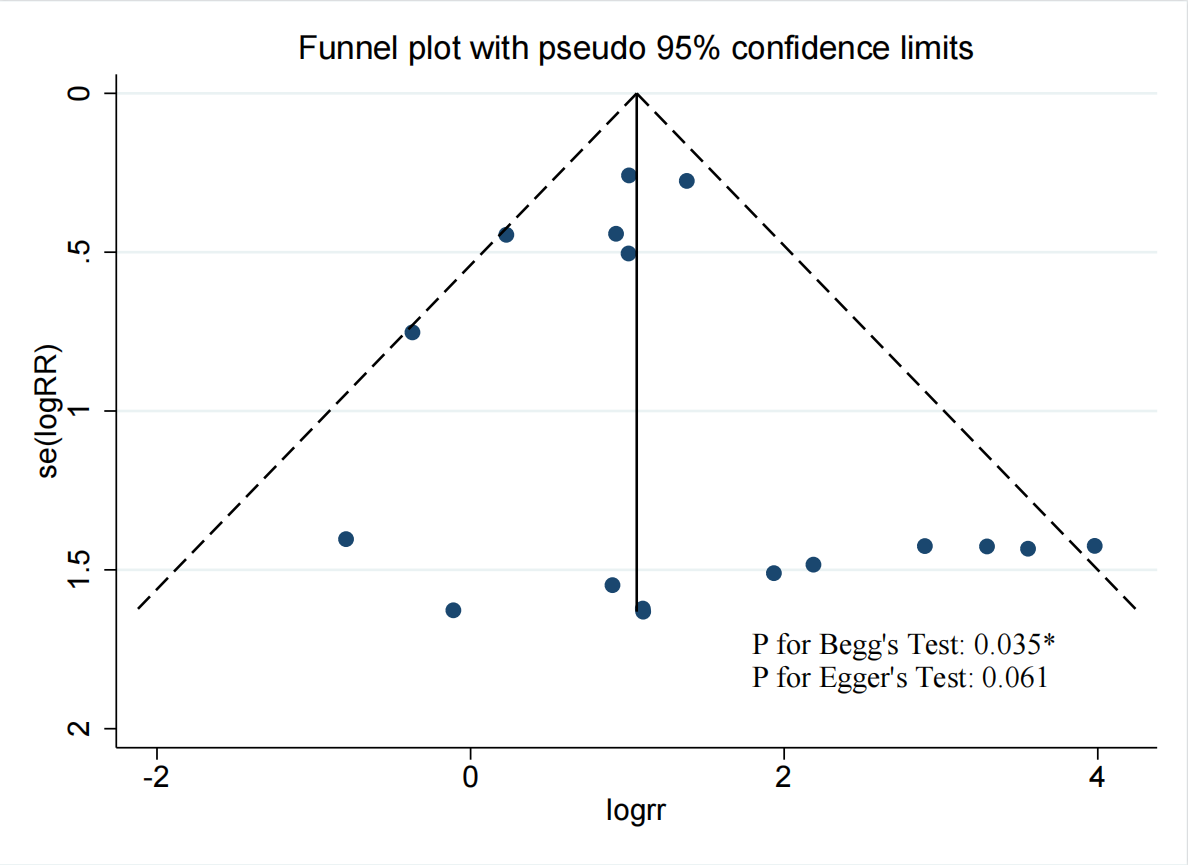


**Supplementary Figure 7. Funnel plot of RCTs with coronary artery disease**
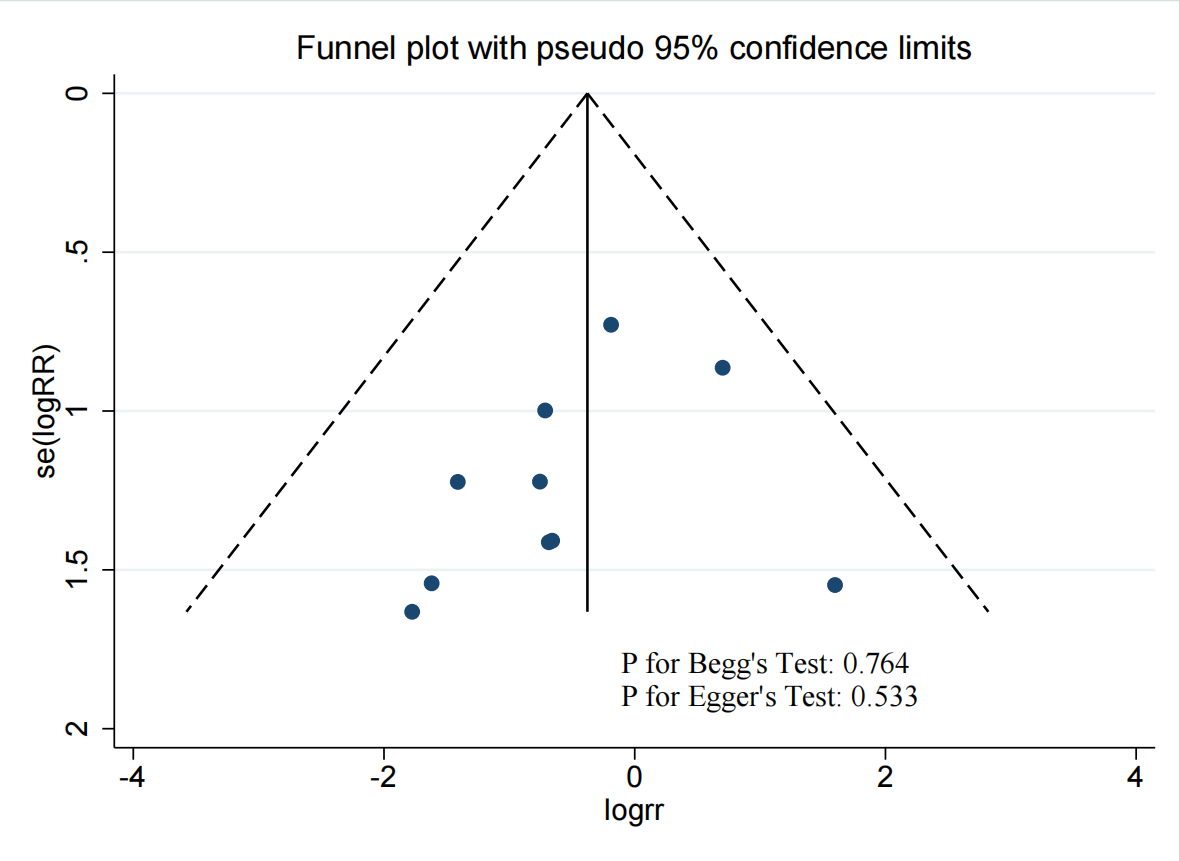


**Supplementary Figure 8. Funnel plot of RCTs with hypertension**
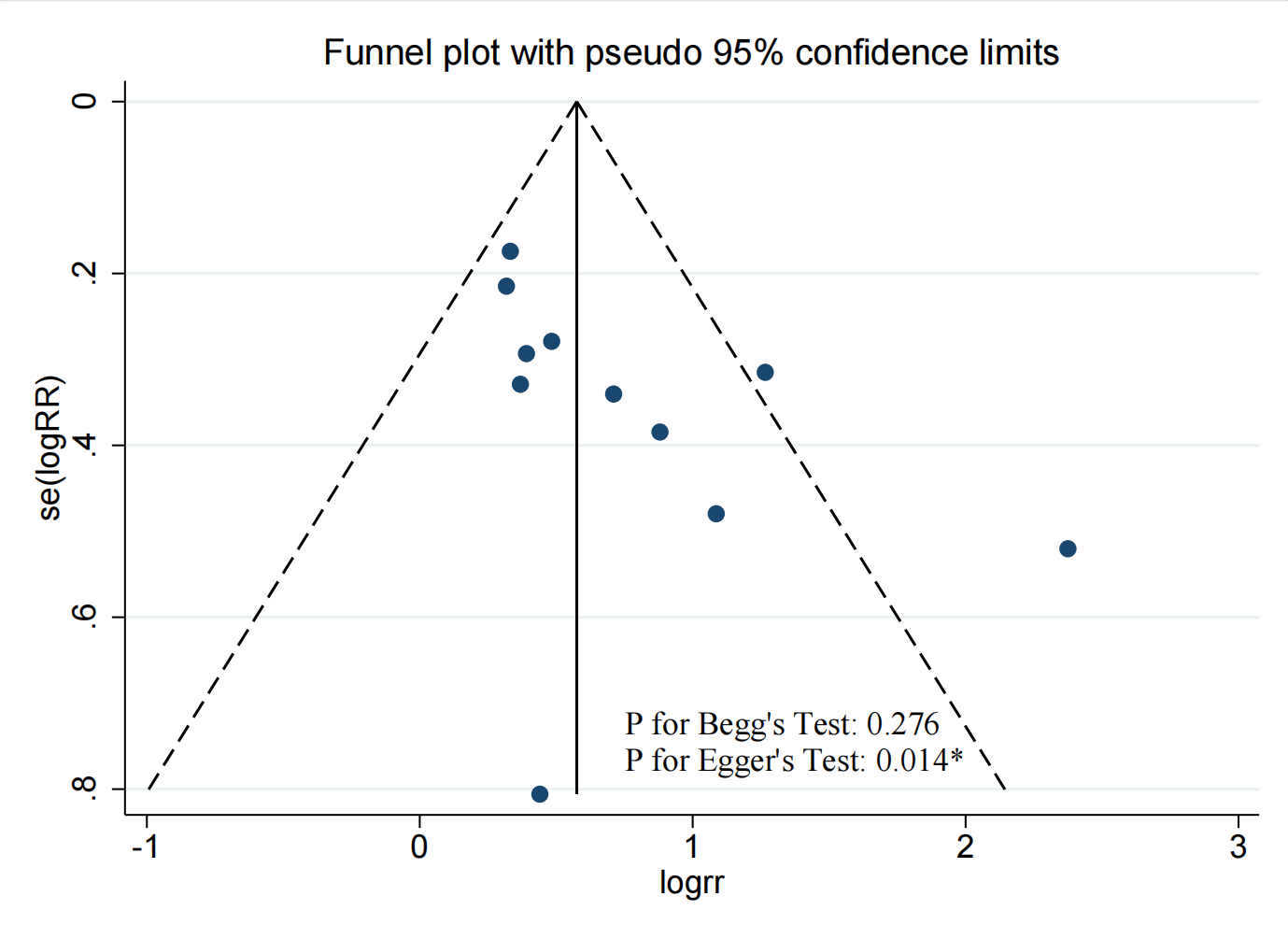


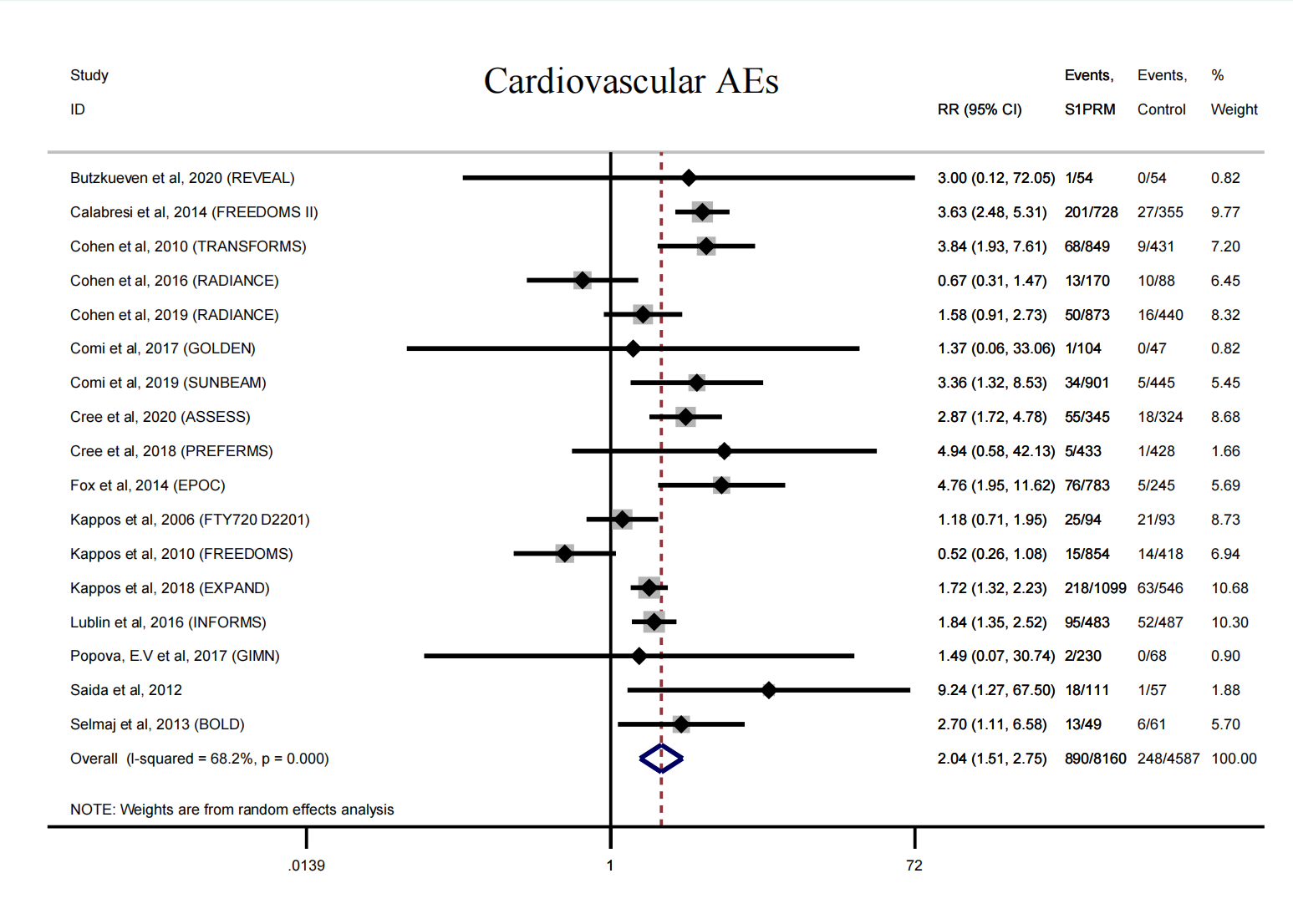
**Supplementary Figure 9. Relative risk of cardiovascular AEs**

**Supplementary Figure 10. Relative risk of cardiovascular AEs**
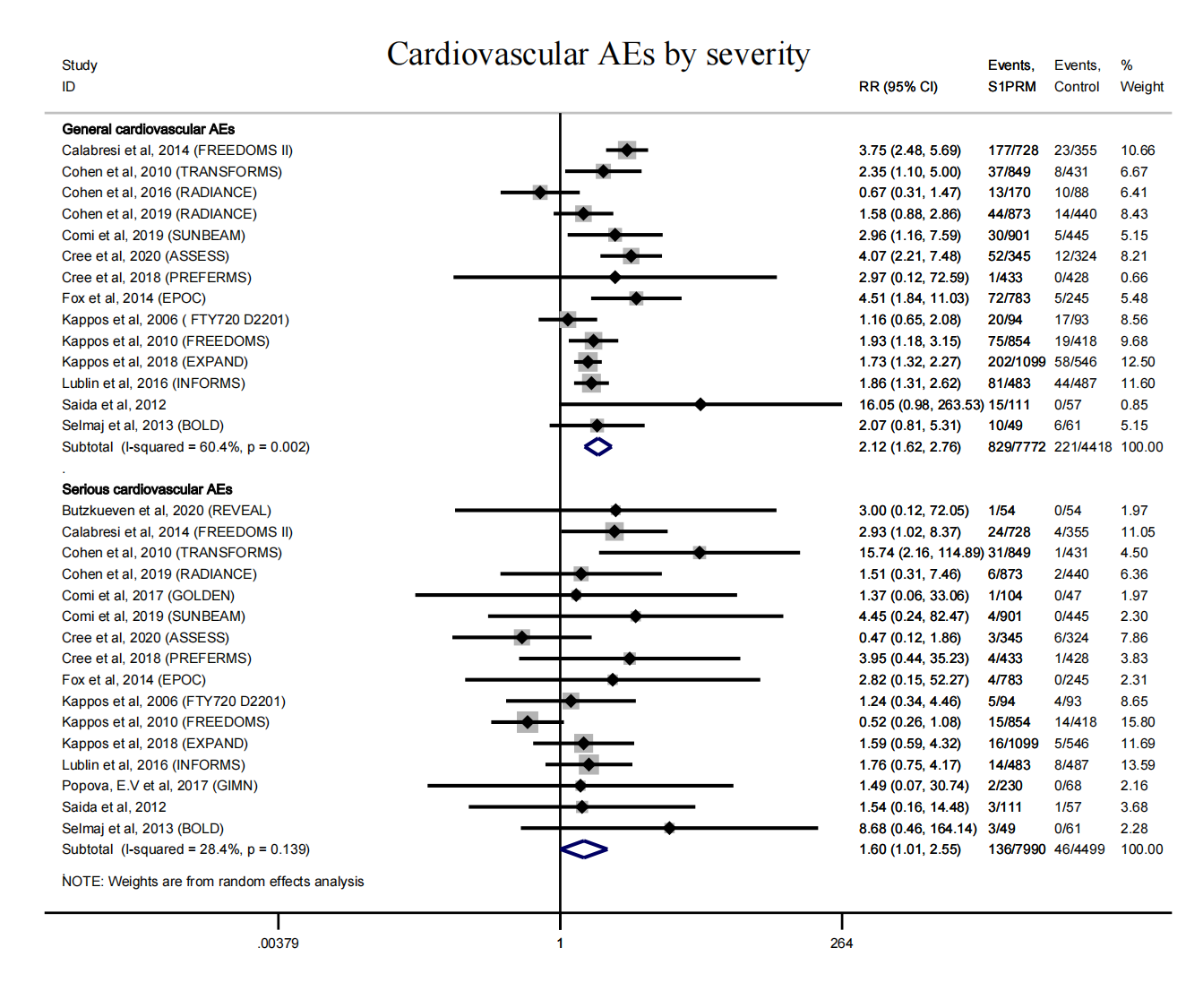
 **by severity**

**
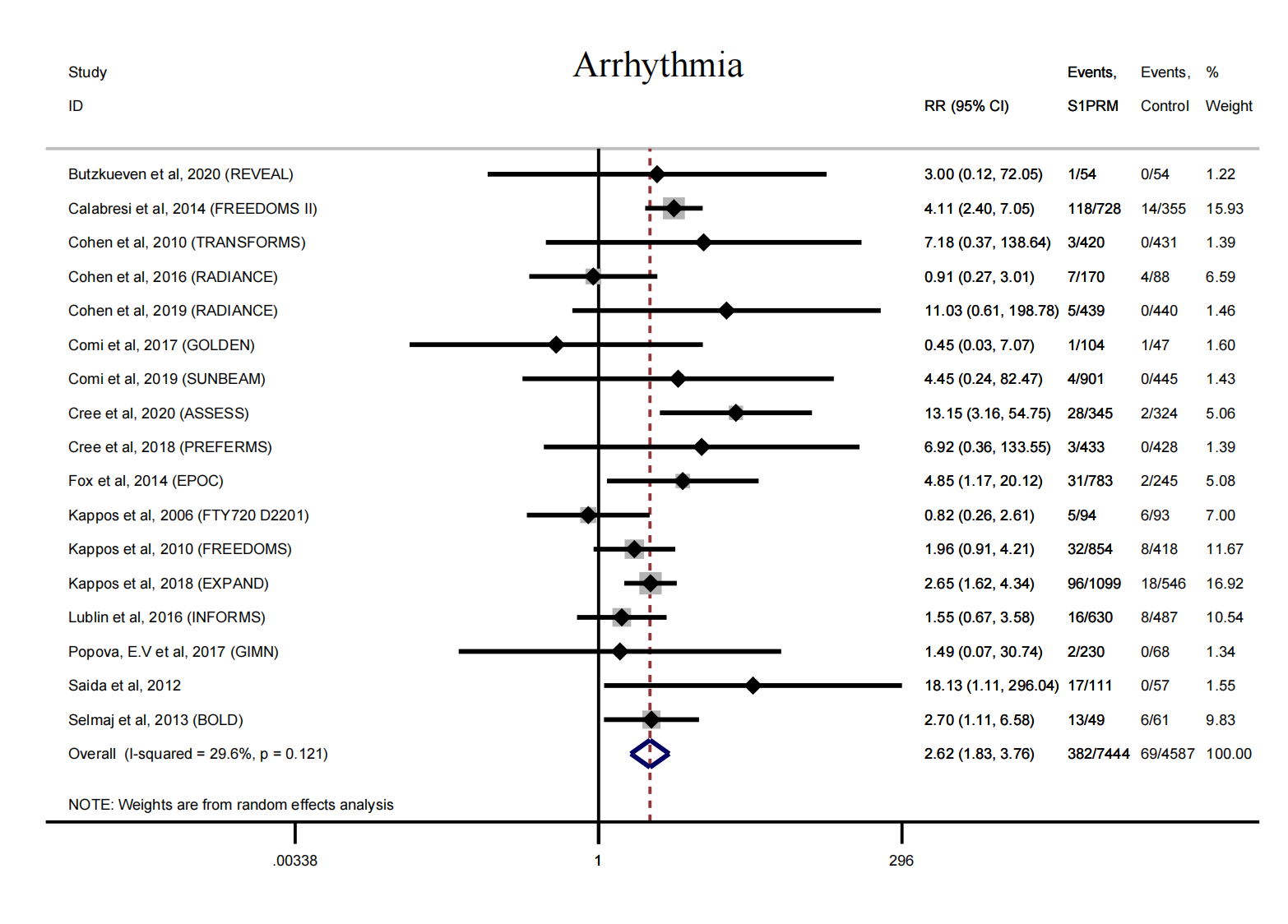
Supplementary Figure 11. Relative risk of arrhythmia**

**
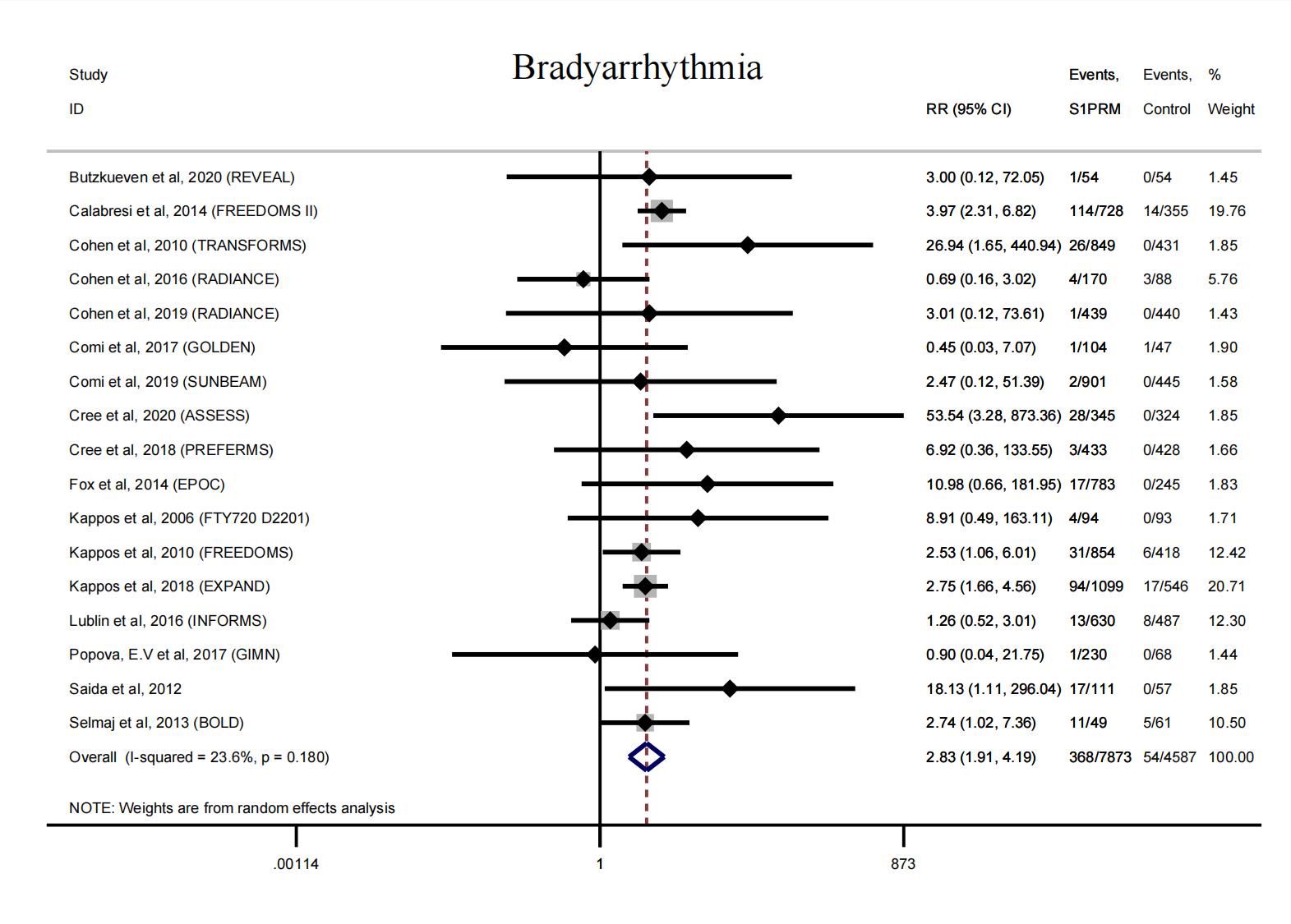
Supplementary Figure 12. Relative risk of bradyarrhythmia**

**
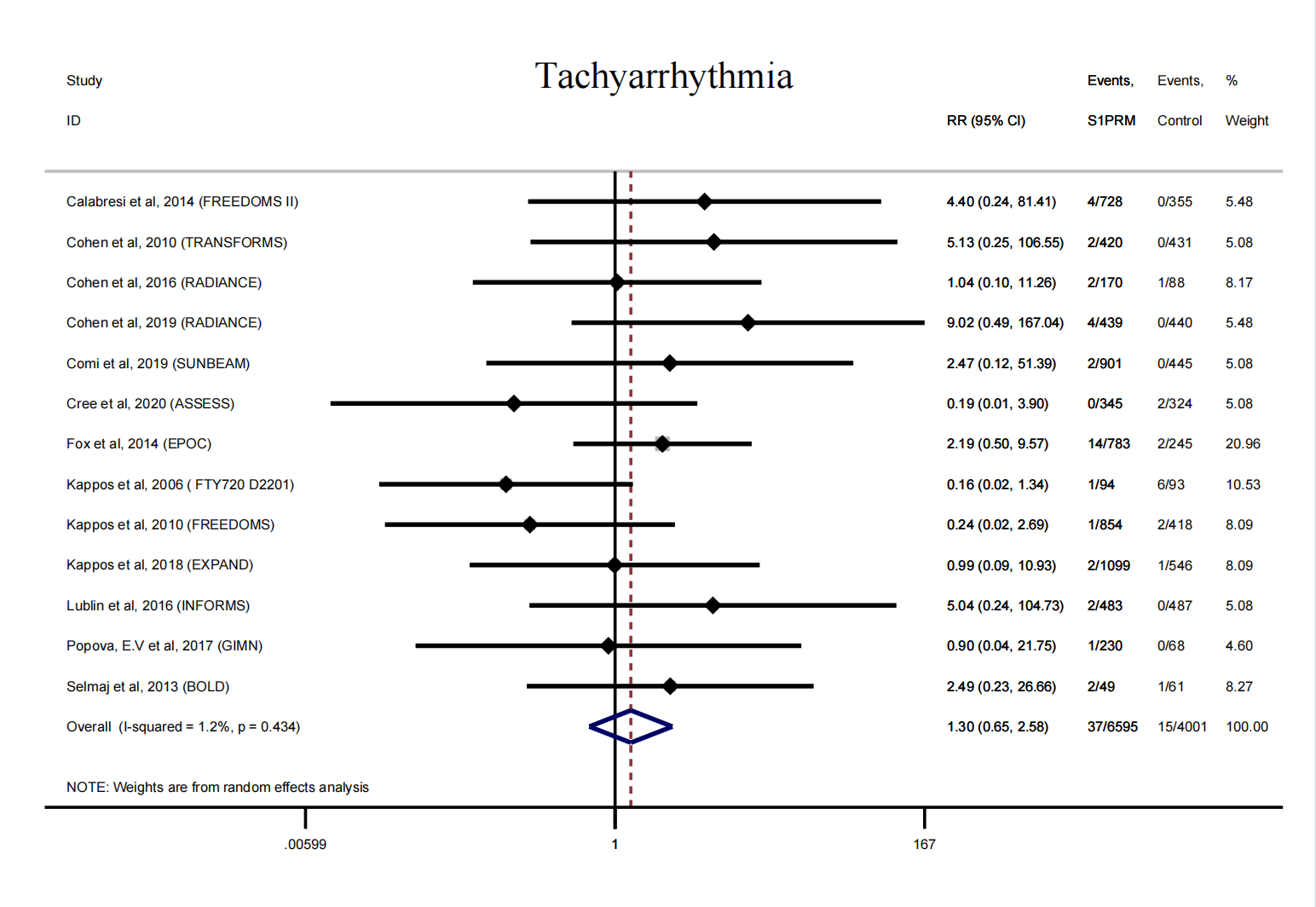
Supplementary Figure 13. Relative risk of tachyarrhythmia**

**Supplementary Figure 14. Relative risk of hypertension**
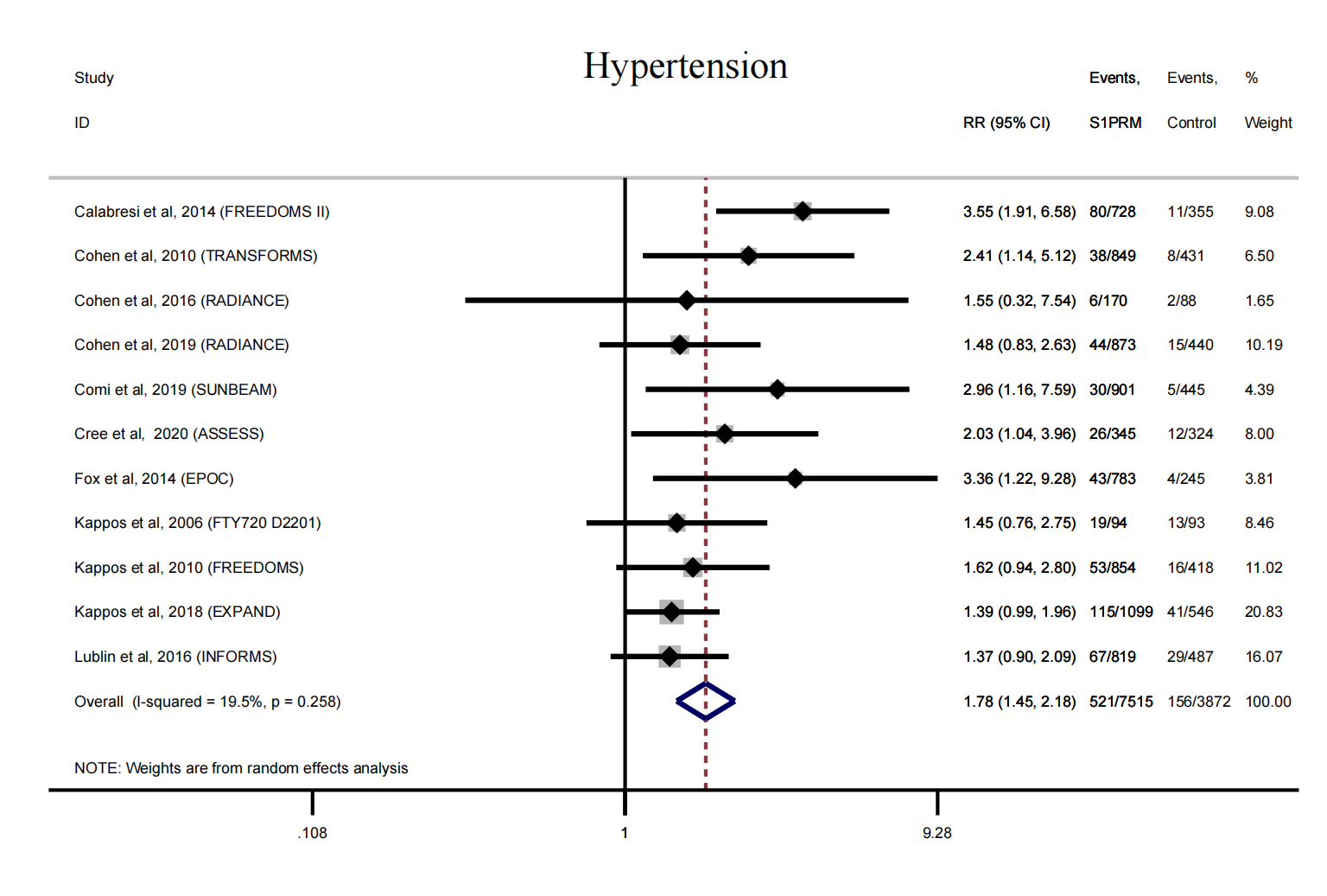


**Supplementary Figure 15. Relative risk of hypotension**
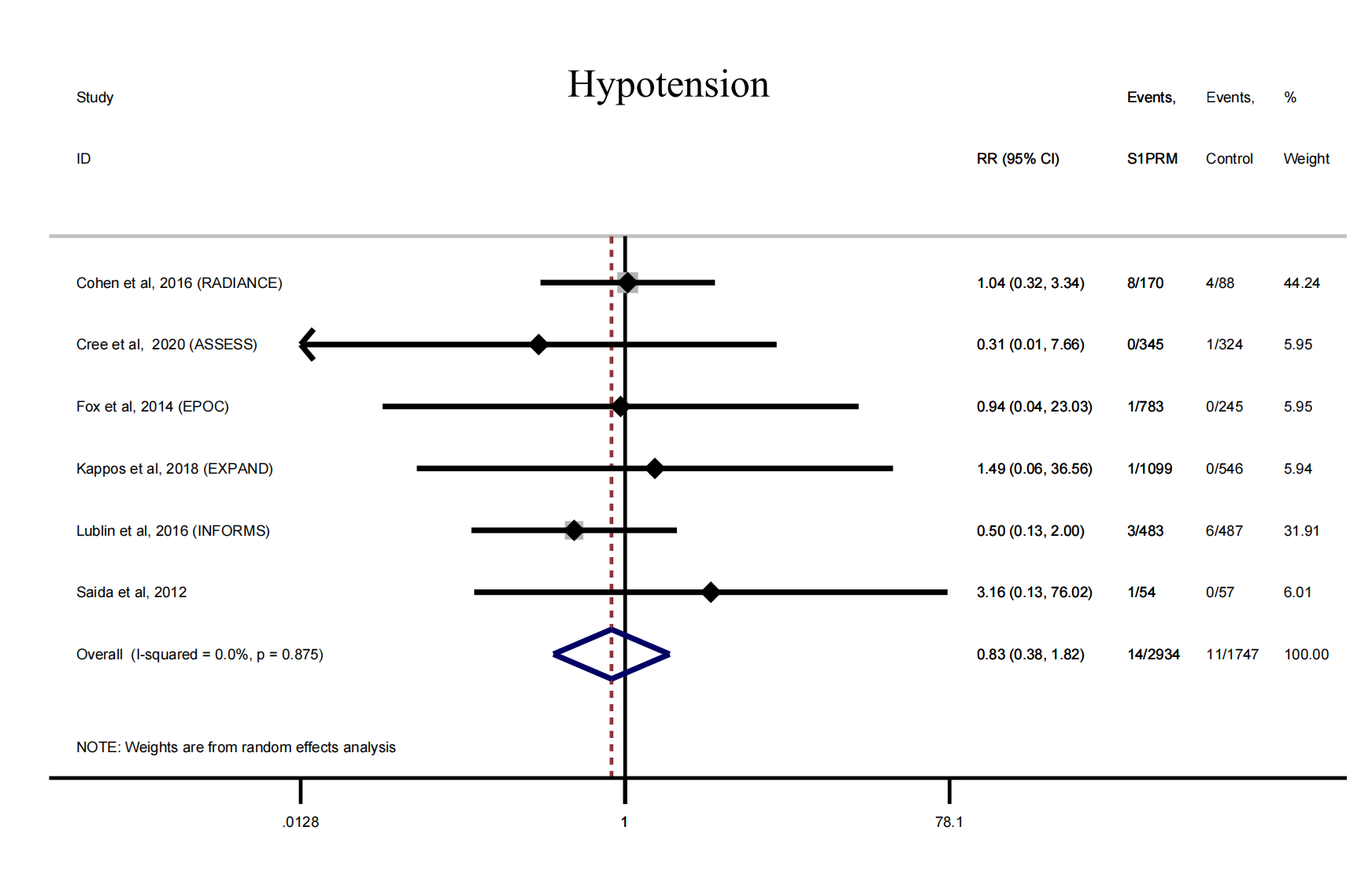


**Supplementary Figure 16. Relative risk of heart failure**
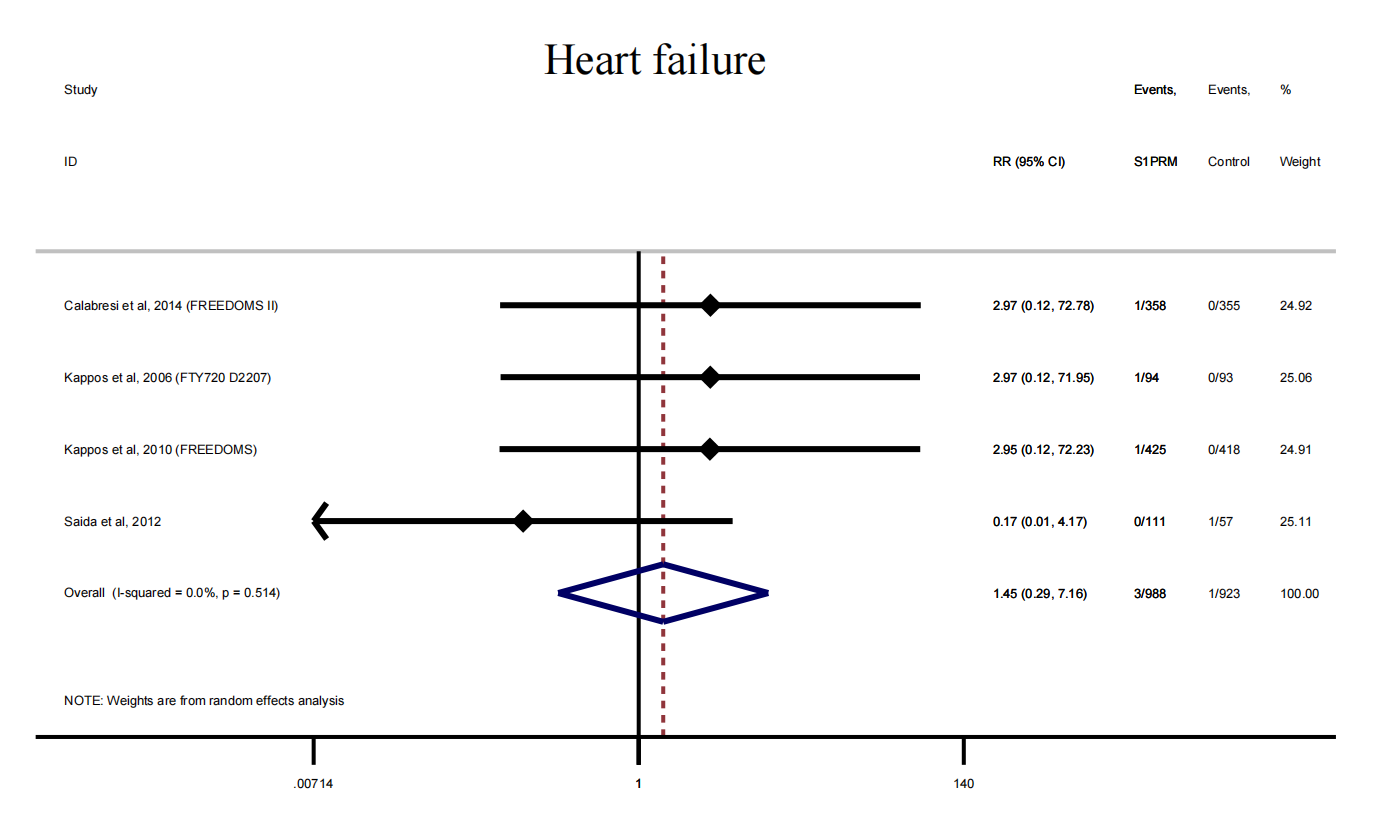


**Supplementary Figure 17. Relative risk of coronary artery disease**
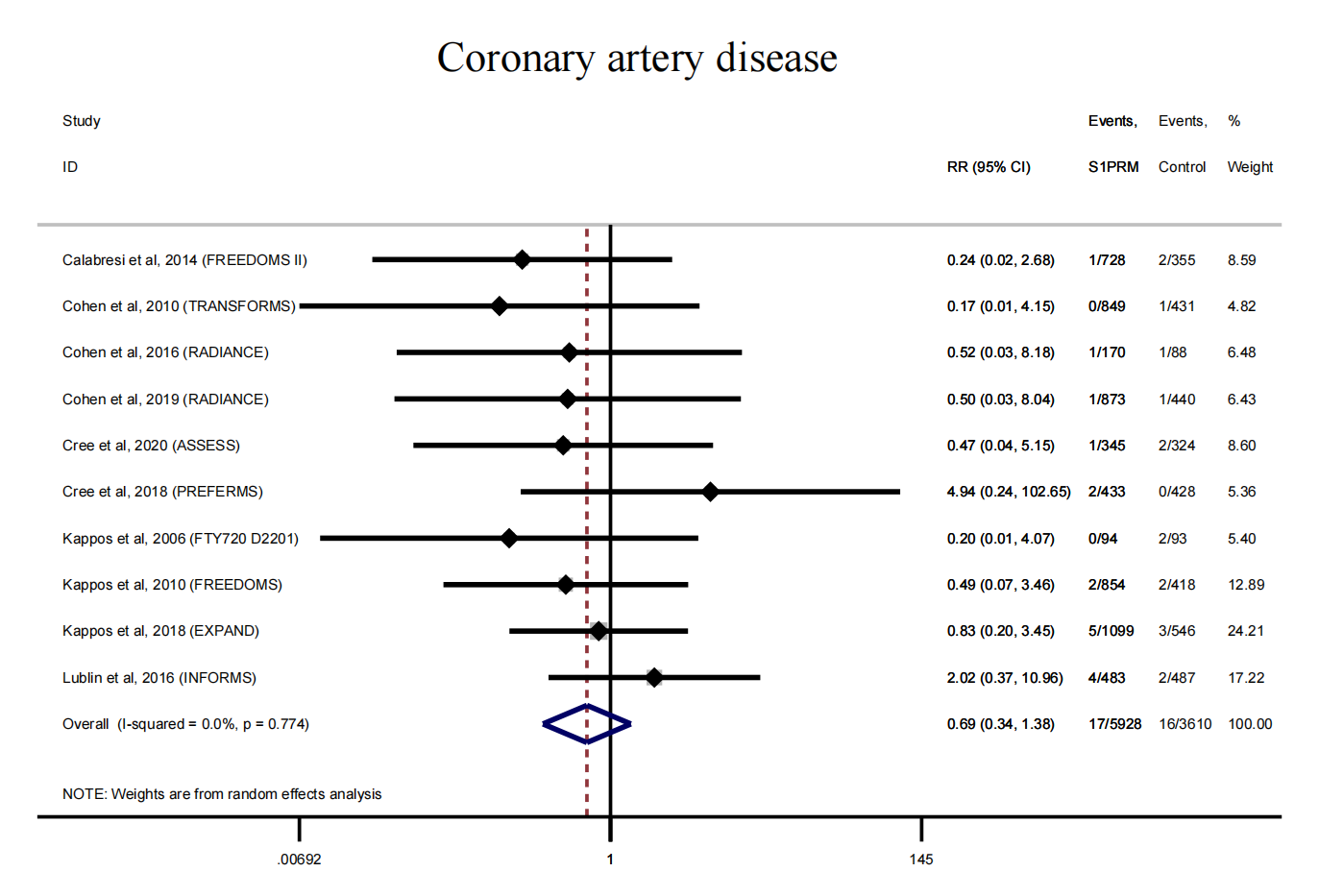


**Supplementary Figure 18. Relative risk of acute coronary syndrome**
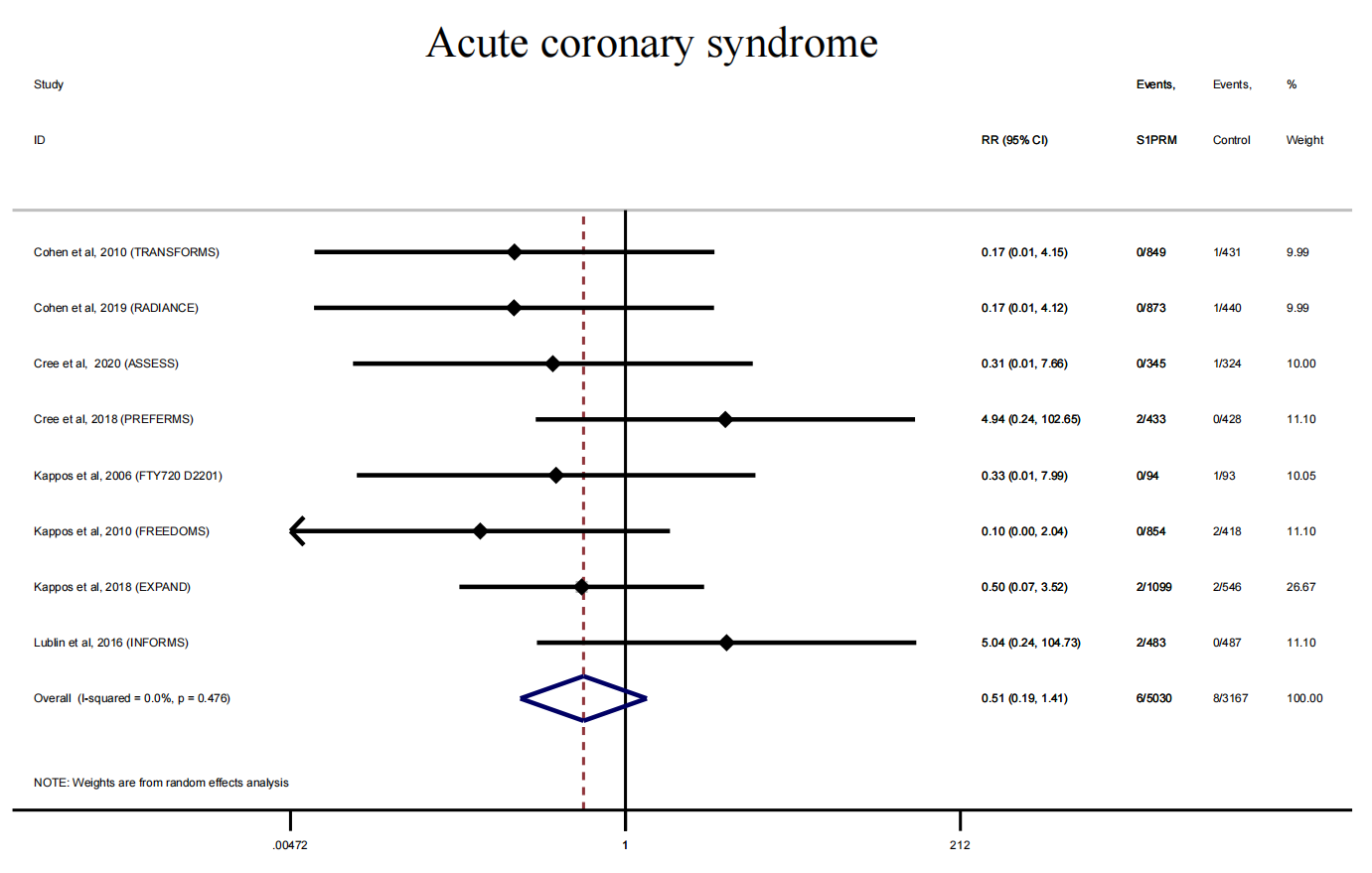


**Supplementary Figure 19. Relative risk of acute coronary syndrome**
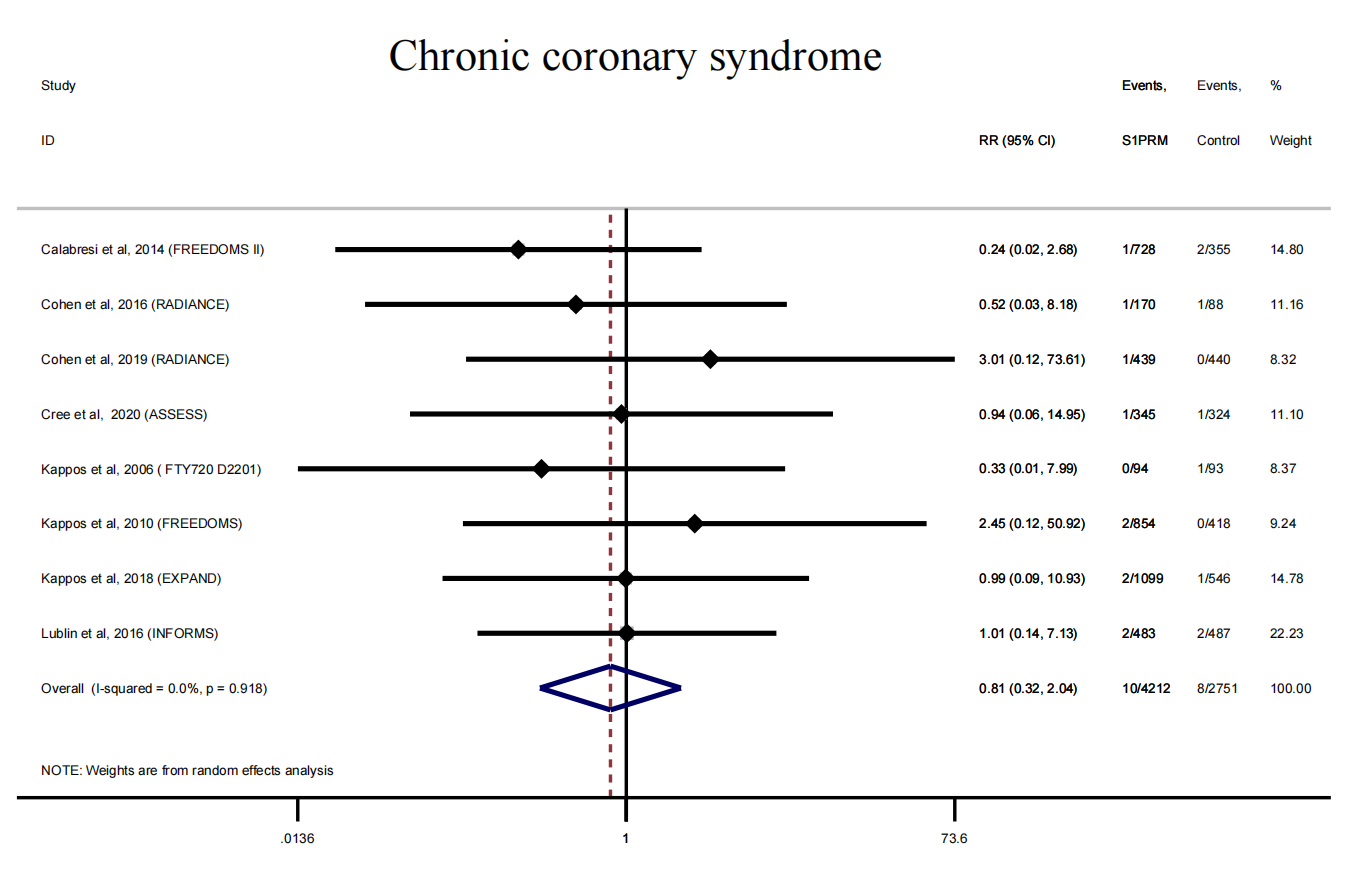


**Supplementary Figure 20. Relative risk of bradyarrhythmia by individual S1PRMs**
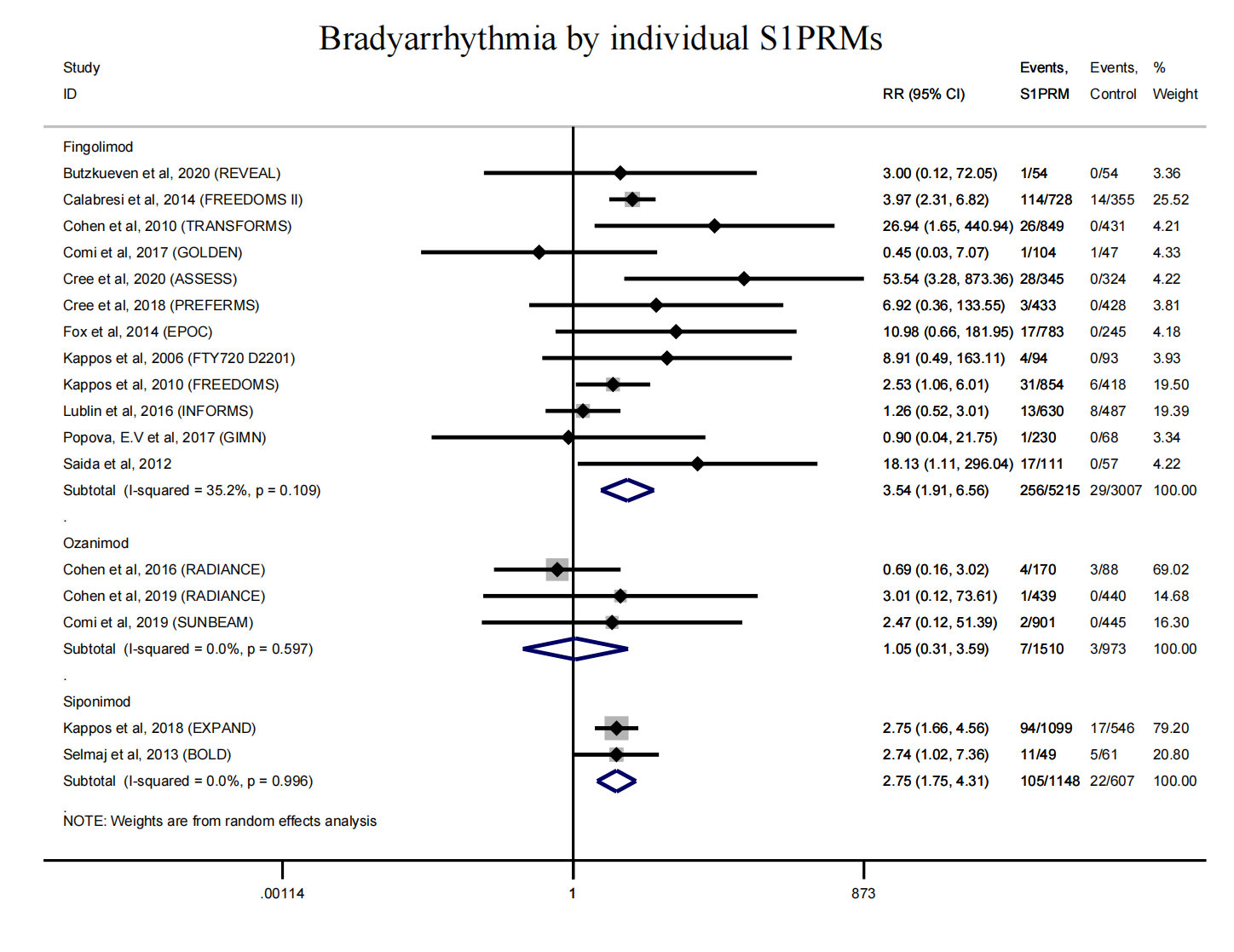


**Supplementary Figure 21. Relative risk of bradyarrhythmia associated with fingolimod by dose**
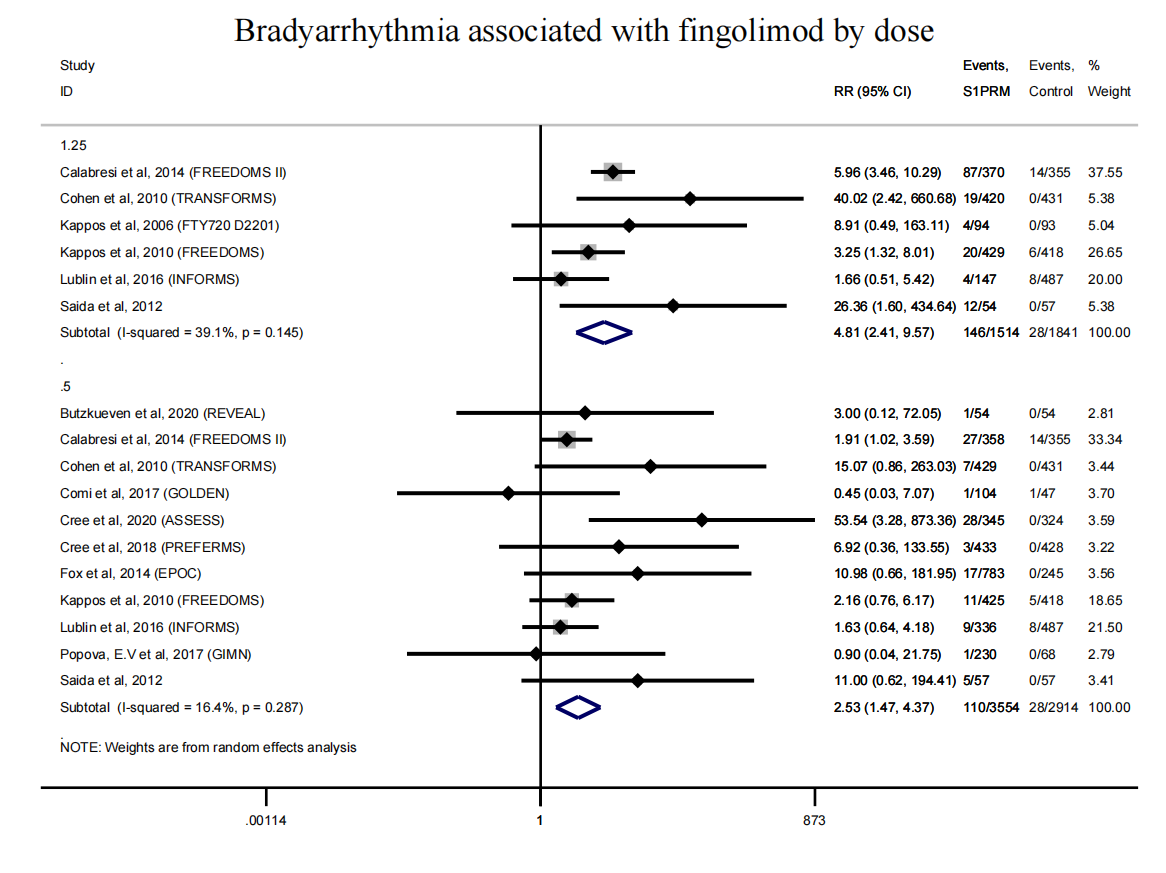


**
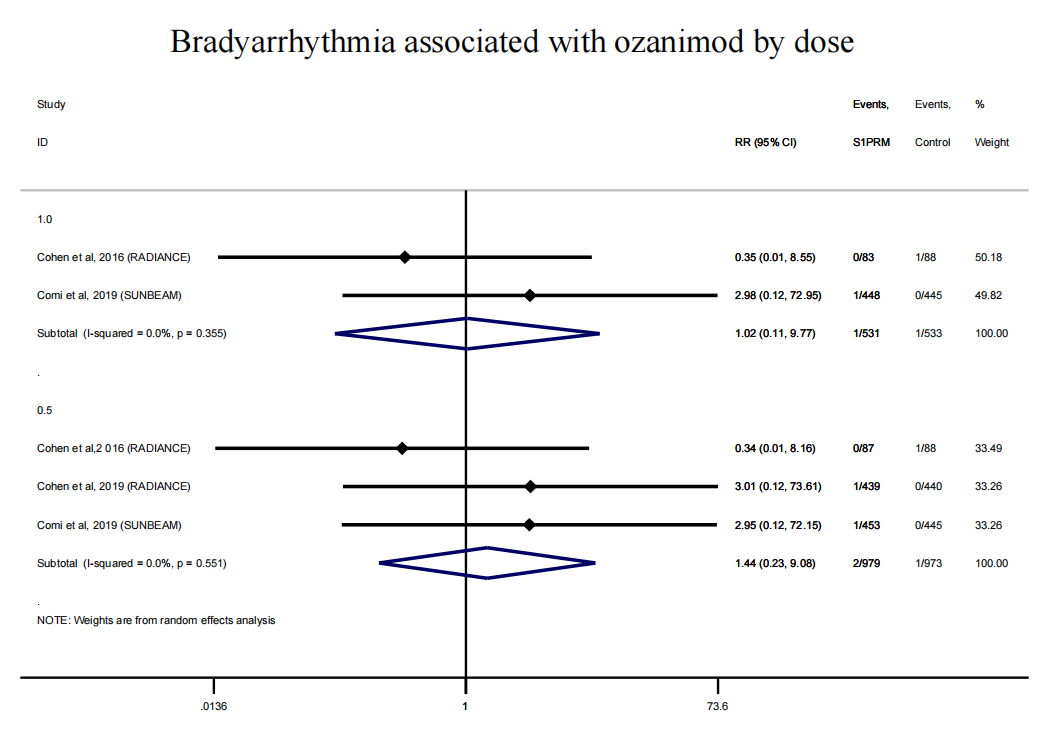
Supplementary Figure 22. Relative risk of bradyarrhythmia associated with ozanimod by dose**

**Supplementary Figure 23. Relative risk of hypertension by individual S1PRMs**
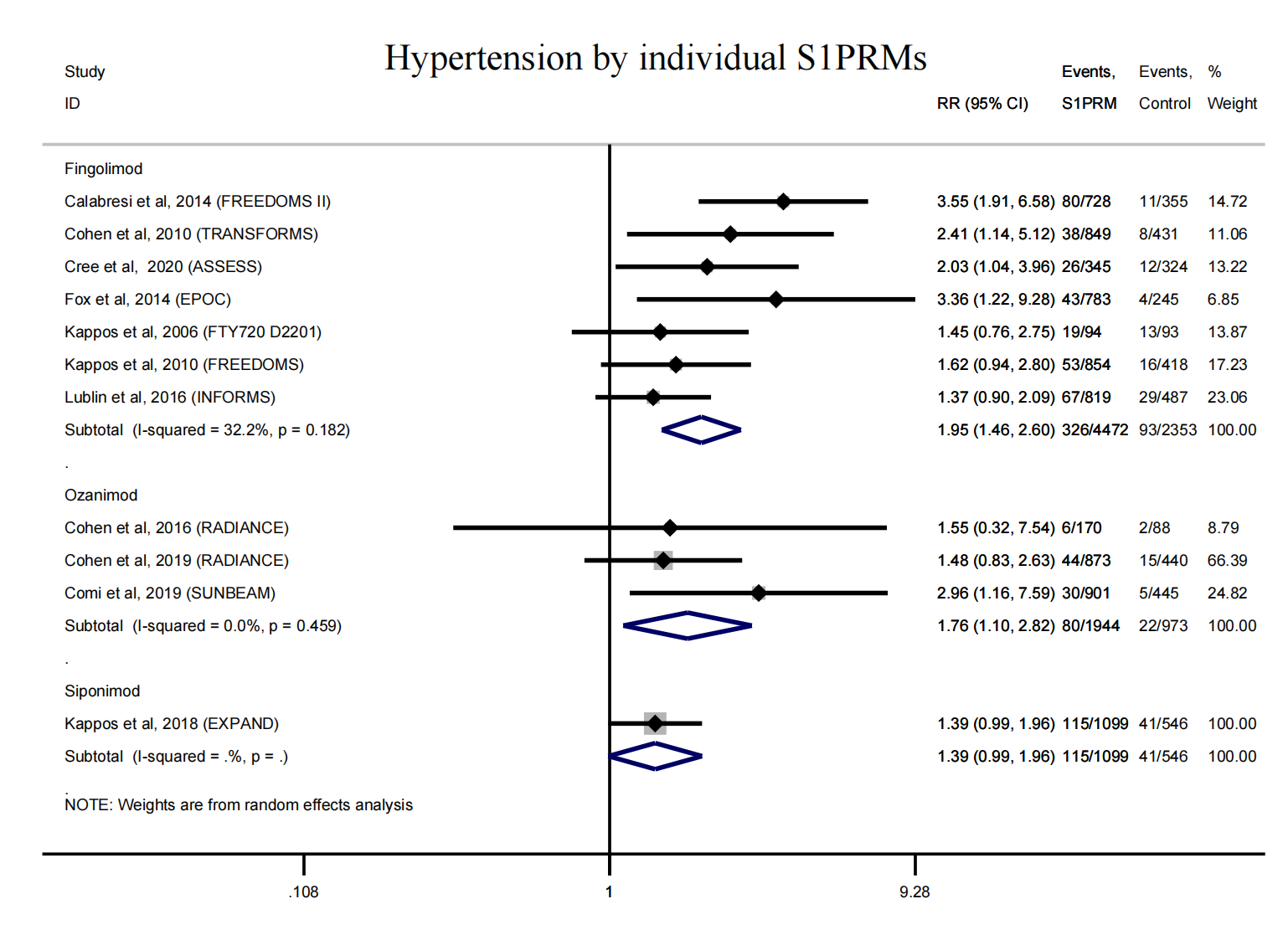


**Supplementary Figure 24. Relative risk of hypertension associated with fingolimod by dose**
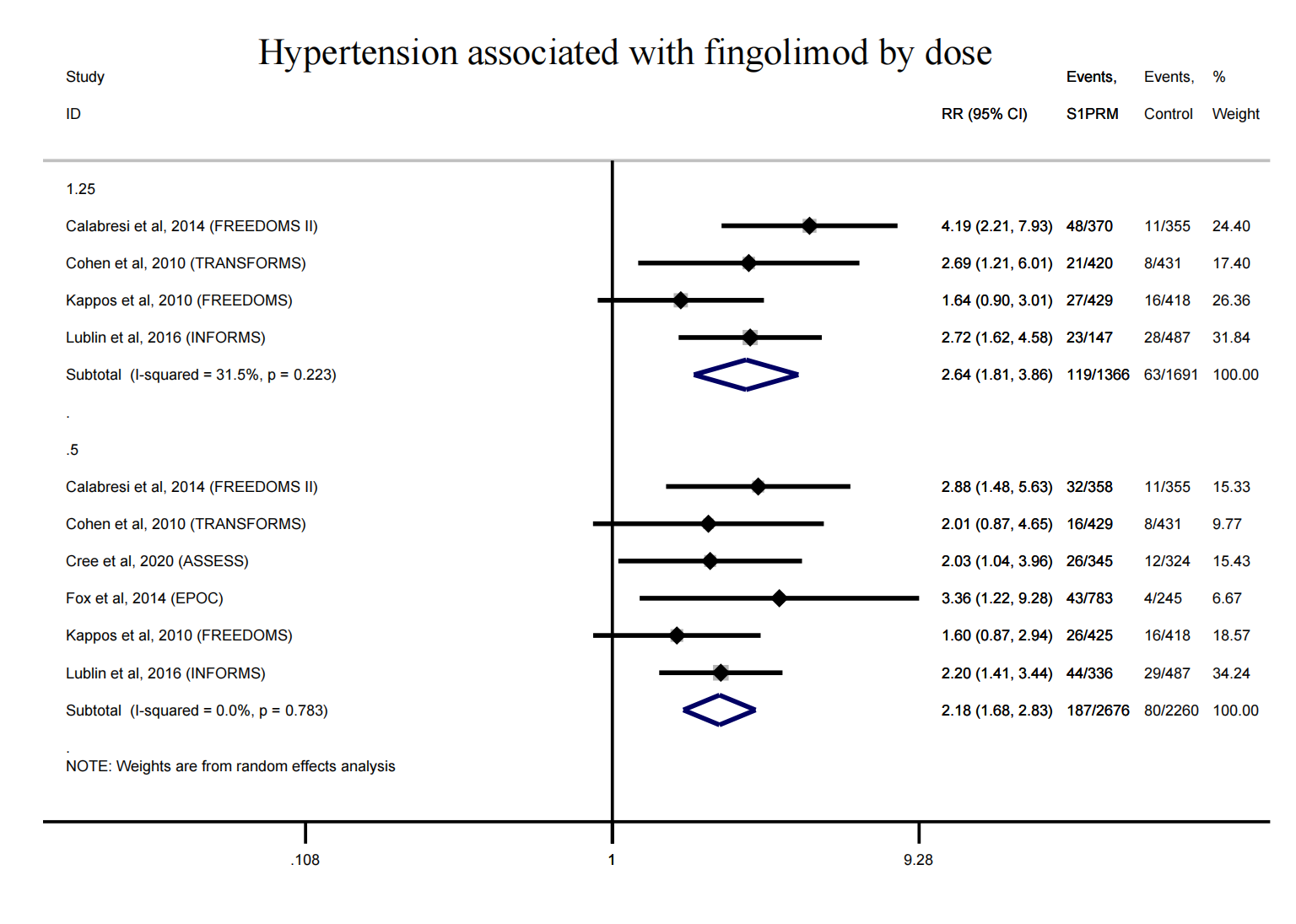


**Supplementary Figure 25. Relative risk of hypertension associated with ozanimod by dose**
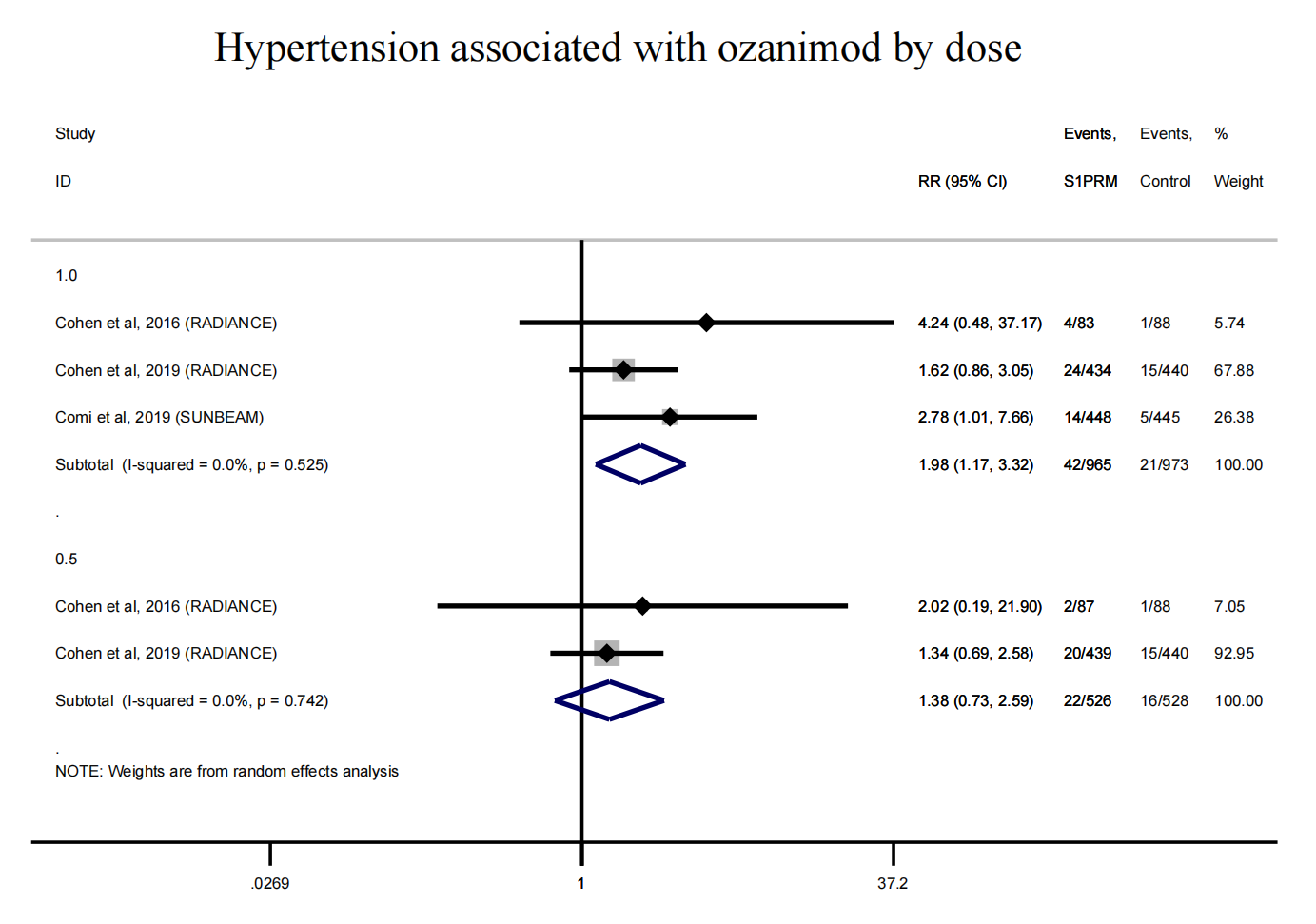


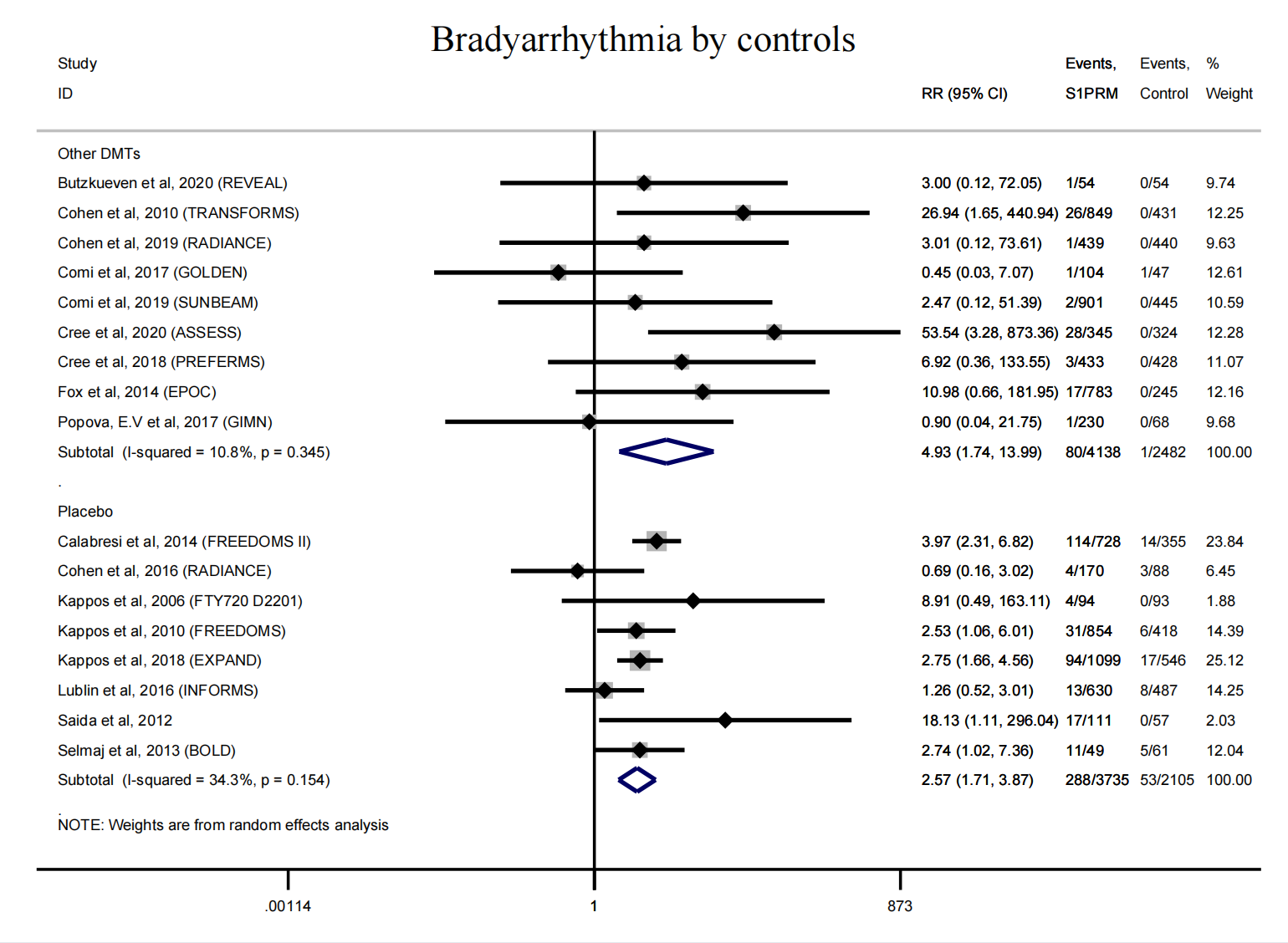
**Supplementary Figure 26. Relative risk of bradyarrhythmia by controls**

**
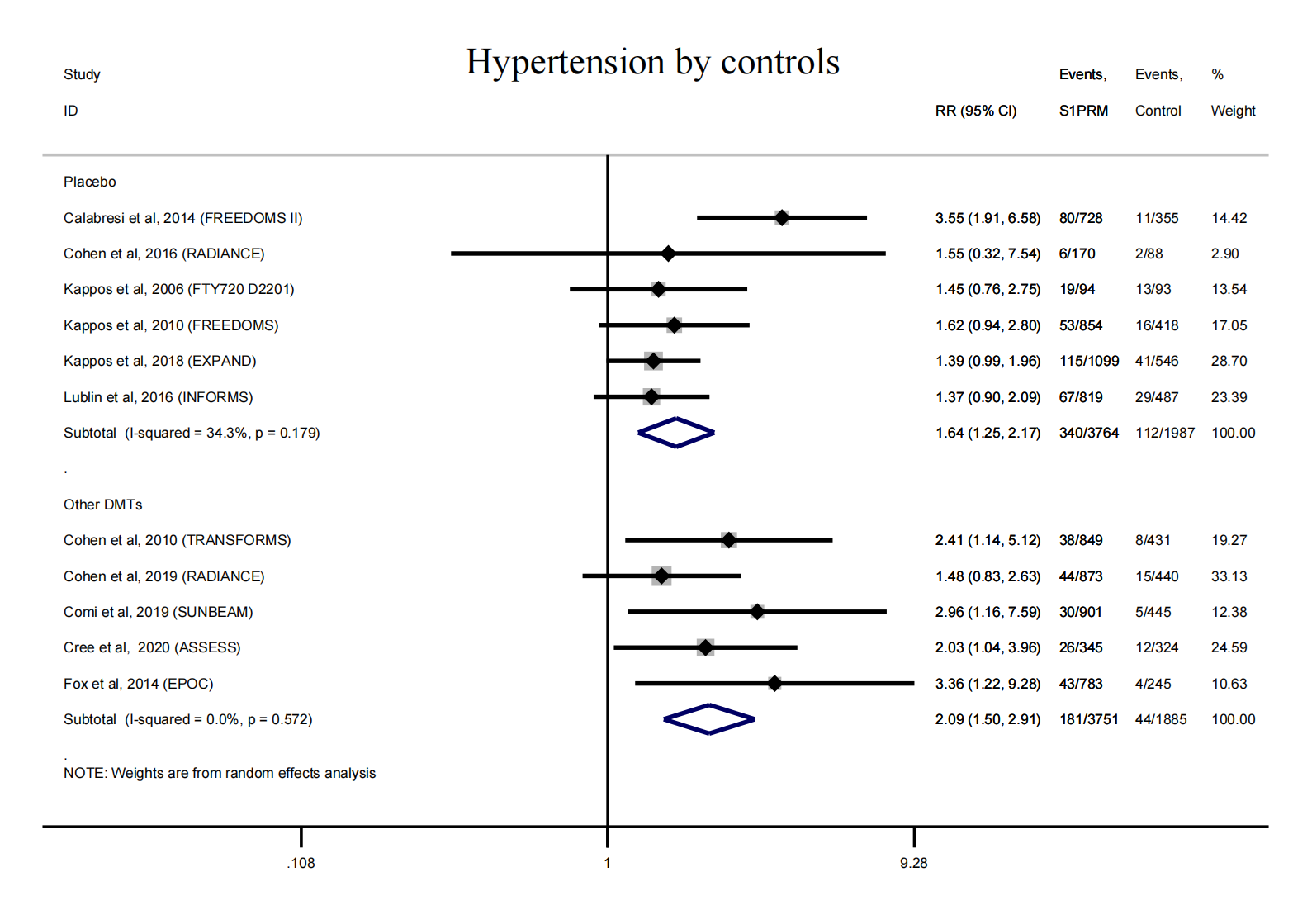
Supplementary Figure 27. Relative risk of hypertension by controls**
